# Are We Filling the Data Void? An Assessment of the Amount and Extent of Plant Collection Records and Census Data Available for Tropical South America

**DOI:** 10.1371/journal.pone.0125629

**Published:** 2015-04-30

**Authors:** Kenneth Feeley

**Affiliations:** International Center for Tropical Botany, Department of Biological Sciences, Florida International University, Miami, Florida, United States of America, and The Fairchild Tropical Botanic Garden, Coral Gables, Florida, United States of America; Cirad, FRANCE

## Abstract

Large-scale studies are needed to increase our understanding of how large-scale conservation threats, such as climate change and deforestation, are impacting diverse tropical ecosystems. These types of studies rely fundamentally on access to extensive and representative datasets (i.e., “big data”). In this study, I asses the availability of plant species occurrence records through the Global Biodiversity Information Facility (GBIF) and the distribution of networked vegetation census plots in tropical South America. I analyze how the amount of available data has changed through time and the consequent changes in taxonomic, spatial, habitat, and climatic representativeness. I show that there are large and growing amounts of data available for tropical South America. Specifically, there are almost 2,000,000 unique geo-referenced collection records representing more than 50,000 species of plants in tropical South America and over 1,500 census plots. However, there is still a gaping “data void” such that many species and many habitats remain so poorly represented in either of the databases as to be functionally invisible for most studies. It is important that we support efforts to increase the availability of data, and the representativeness of these data, so that we can better predict and mitigate the impacts of anthropogenic disturbances.

## Introduction

Big problems call for big ecology. Big ecology needs big data.

There is a rapidly increasing need for large-scale studies in order to predict and mitigate the effects of large-scale conservation threats, such as deforestation and climate change [1,2]. For example, various studies have used massive collections of natural history records, range maps, and census plot data to estimate patterns of biodiversity across continental-scale areas and to predict how diversity will be impacted under different scenarios of climate change and habitat loss [3–10]. These large-scale studies use “big data”—data that is generally beyond the scope of what can be collected by individual researchers or through individual projects [2]. As such, these studies often depend heavily on extensive and expansive collations of datasets that are standardized and made available through collaborative networks or data clearinghouses.

One of the most important clearinghouses for biogeographic and natural history data is the Global Biodiversity Information Facility (GBIF; http://www.gbif.org/). Indeed, since it contains copious amounts of data, is easy to use, is compatible with popular biogeographic methods (e.g., species distribution modeling) and is entirely open access, GBIF has rapidly become one of the most widely-used and important resources in ecology, biogeography, and conservation biology since its launch in 1997. According to their own statistics, GBIF data has been used in nearly 900 peer-reviewed scientific publications to date.

While species occurrence databases, such as those linked through GBIF, are clearly a powerful and important resource, many studies have pointed out its potential limitations for biogeographic and ecological studies [11]. These limitations can be due to problem with data quality. For example, collections data are prone to taxonomic and georeferencing errors [12,13] and may suffer from biases in the taxonomic and spatial representativeness of the samples [14–18] due in part to the understandable tendency of collectors to focus their efforts in accessible areas and areas with well-established logistical and intellectual infrastructures [19–21].

Another potential limitation of occurrence databases is simply insufficient data quantity [14]. In 2011, Feeley and Silman, reported on the extreme paucity of collections data in GBIF (and a similar database for Brazil named SpeciesLink; http://splink.cria.org.br/) for tropical plant species. Specifically, using data downloaded in 2009 they estimated that only about 65% of tropical plant species were represented by any available geo-referenced collections and that of the represented species, only about 8% or 0.5% (approx. 5% or 0.3%, respectively, of all tropical plant species) had enough available records to be used in species distribution models or other analyses requiring 20 or 100 minimum samples, respectively [22]. Given the dominant role that GBIF (and at the time, SpeciesLink) plays in distributing natural history records, this lack of data from the tropics was considered a major constraint on studies of tropical species and diversity. Perhaps more troubling, the lack of available records from the tropics was considered symptomatic of a more general lack of knowledge about the distribution and ecology of most tropical species as well as a lack of knowledge about the composition and structure of vast expanses of the tropics. Feeley and Silman referred to this lack of knowledge as the “data void” [22].

Since Feeley and Silman published their study in 2011, GBIF has continued to grow and the amount of data available from all regions, including the tropics, has greatly increased. This growth has been due to ongoing collection efforts, the digitization of additional pre-existing records, and inclusion of new datasets into GBIF. There have also been laudable efforts at data standardization and cleaning (e.g., the Taxonomic Name Resolution Service, TNRS; http://tnrs.iplantcollaborative.org/) which affects the number of species represented in the dataset (in most cases decreasing the number of species through the elimination of synonyms and false species created by spelling errors) and the number of records available per species (generally increasing the number of records per species through the combination of records formerly assigned to different species names).

While clearly important, natural history and occurrence records such as those provided by GBIF are inherently limited in their utility. For example, the geo-referenced data contained in natural history records can be used to map species ranges in relation to large climatic gradients, but they provide no information about local patterns of occurrence, species abundances, alpha diversity, or community composition. These types of patterns are better assessed through analyses of intensive plot inventories or censuses. Over the past several years there have been notable attempts to collate and standardize tropical forest inventory data (i.e., plot data) through collaborative networks. For example, the Amazon Tree Diversity Network (ATDN; http://web.science.uu.nl/Amazon/atdn/) and the RAINFOR Amazon Forest Inventory Network (http://www.rainfor.org/) have each compiled data from hundreds of pre-existing forest inventory plots in the Amazon basin supplemented with subsequent installations of new plots in targeted areas. These and other networks of plot census data are being used to look at large-scale patterns in forest structure and composition across the Amazon [5,6,23–26].

Here, I asses the availability of occurrence and census data from the tropics and examine how this availability has varied through time as well as how it varies through space. Specifically, I quantify the amount of occurrence data available through GBIF for plant species and from different habitats in tropical South America and examine how data availability has changed through time. I also analyze the spatial and habitat distribution of South American forest census plots as represented in several of the most prominent plot networks. The goal of this study is to characterize the state of data availability for tropical species and habitats of South America and to evaluate the rate that we are, or are not, filling the data void. By understanding where our data limitations are, we can better assess the generalizability of the results emerging from analyses of the existing data and direct future studies to help reduce these data limitations.

## Methods

### Data

All records for plant species (kingdom plantae) occurring in the tropical latitudes of South America were downloaded from the GBIF data portal on February 1^st^ 2014 (see S1 File for list of contributing databases and herbaria). The records were screened to exclude those without geo-referencing information or with obvious errors in geo-referencing data (i.e., flagged by GBIF for data quality issues or with coordinates occurring in ocean or in areas outside of South America). All of the species names listed with the collection records were then verified using the online Taxonomic Name Resolution Service (TNRS) to remove synonyms and correct for spelling errors. Duplicate records were removed by screening for records with identical species names and collection coordinates.

I collated the locations of inventory plots as published online and in published articles for five of the largest and most prominent South American census plot networks: RAINFOR, ATDN, Forestplots.net (https://www.forestplots.net/; [6]), the Smithsonian Institute’s Center for Tropical Forest Science (CTFS; http://www.ctfs.si.edu/), and the Red de Bosques (http://www.condesan.org/redbosques/). Many individual census plots are members of multiple networks so all duplicate records were identified and removed.

In order to assess the representation of different habitats in the herbarium and plot datasets, I classified the habitats of Tropical South America according to WWF ecoregions (http://www.worldwildlife.org/publications/terrestrial-ecoregions-of-the-world; [27]). In addition, I divided tropical South America into discrete climatic zones on the basis of mean annual temperature (MAT) and total annual precipitation (TAP). Estimates of MAT and TAP were based on “current” (mean of 1960–1990) conditions according to the WorldClim database (http://www.worldclim.org/ [28]). Climatic zones were characterized as those areas having different combinations of MAT = 0–2°C, 2–4°C, 4–6°C, 6–8°C, 8–10°C, 10–12°C, 12–14°C, 14–16°C,16–18°C, 18–20°C, 20–22°C, 22–24°C, 24–26°C, 26–28°C, 28–30°C; and TAP = 0–500mm, 1000–1500mm, 1500–2000mm, 2000–3000mm, 3000–4000mm, 4000–6000mm, and >6000mm.

### Analyses

To calculate the change in data availability in GBIF through time, I tallied the number of records available in each year from 2007 (when GBIF launched) to the end of 2013. I then tallied the number of records available per species in each year. I calculated and mapped the average density of records (no. of records per km^2^) per 0.5 x 0.5 longitude/latitude degree cell and how this record density has changed through time. Using the collated list of plot locations, I measured the straight line distance of all possible 30 arc second grid cell centers in tropical South America to the closest census plot. Finally, I calculated and mapped the average density of collections and census plots within each of the WWF ecoregions and within each of the climatic zones as defined above.

## Results

**Fig 1:**
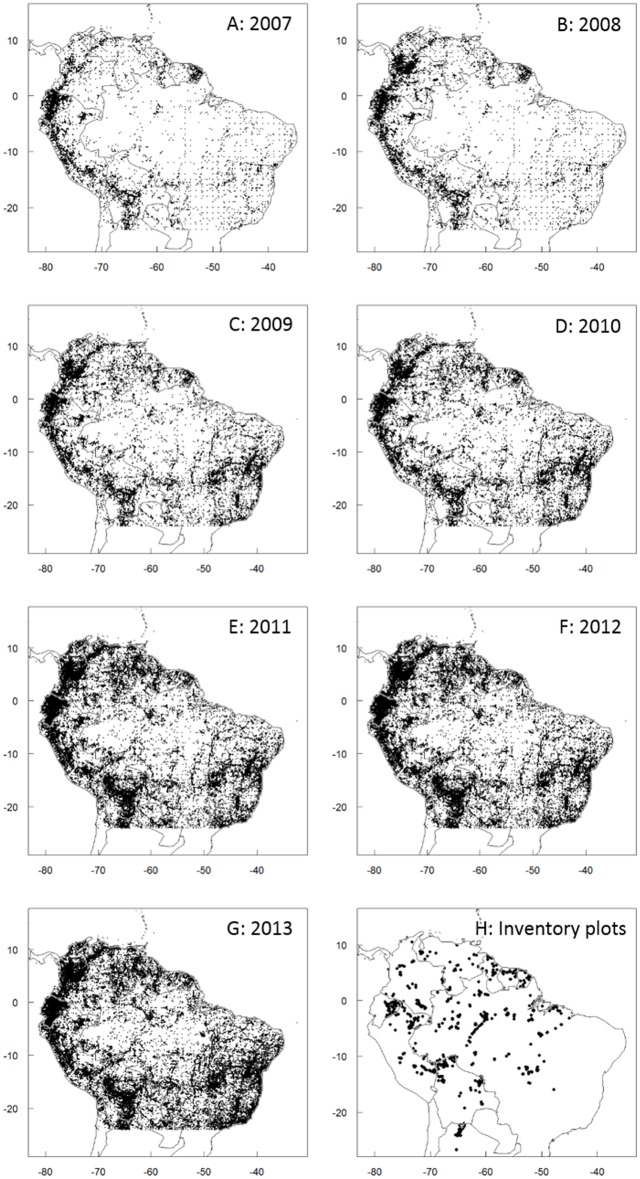
Locations of occurrence records and census plots in tropical South America. Maps showing the location of plant occurrence records available through GBIF each year from 2007 to 2013 (Panels A-G, respectively) and the location of the networked vegetation census plots included in the analyses (Panel H).

**Fig 2:**
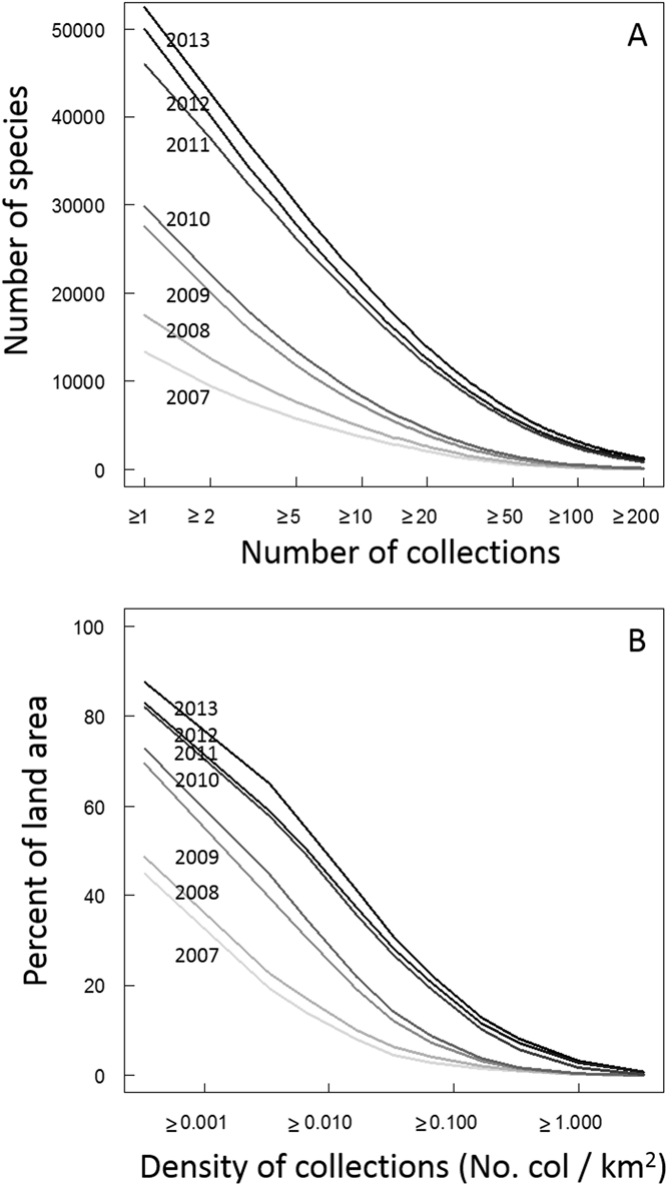
Number of occurrence records per species and density of collection in tropical South America. Panel A shows the cumulative number of occurrence records per plant species of tropical South America available through GBIF in 2007–2013. Panel B shows the cumulative mean density of occurrence records (no. per km^2^) available through GBIF for tropical South America in 2007–2013.

The number of georeferenced plant records available through GBIF for tropical South America has increased rapidly since 2007 (Table 1 and Fig 1a–1g), as has the number of represented species and the number of collections available per species (Table 1 and Fig 2a). Of note is the fact that most of this increase is due to the inclusion of additional pre-existing records rather than new collections. For example, of the 177,925 records added to GBIF in 2013, only 2,910 (1.5%) were of collections that were actually made in 2013. The largest increase in data availability came between 2010 and 2011 when the number of records increased by nearly 300%; this increase was driven in large part by the incorporation of SpeciesLink data into GBIF.

**Table 1:** The availability of plant collections data through the Global Biodiversity and Information Facility (GBIF).

Year	No. of collections	No. of species	Mean no. of collections per species*	Median no. of collections per species*	Mean no. col / km^2^	Median no. col / km^2^	% of area with zero collections
2007	164214	13339	3.15 (12.40)	0 (3)	0.01	0.00	54.81
2008	206218	17454	3.95 (12.93)	0 (3)	0.02	0.00	51.25
2009	331856	27532	6.36 (12.15)	1 (3)	0.02	0.00	30.91
2010	386042	29824	7.40 (13.04)	1 (4)	0.03	0.00	27.50
2011	1098386	46056	21.05(23.98)	4 (6)	0.08	0.01	18.50
2012	1638600	50026	31.40 (32.92)	5 (6)	0.12	0.01	17.73
2013	1816525	52432	34.81(34.81)	6 (6)	0.13	0.01	13.14

* the values in parentheses indicate the mean or median number of collections per species if only the species represented in that year are included.

**Fig 3:**
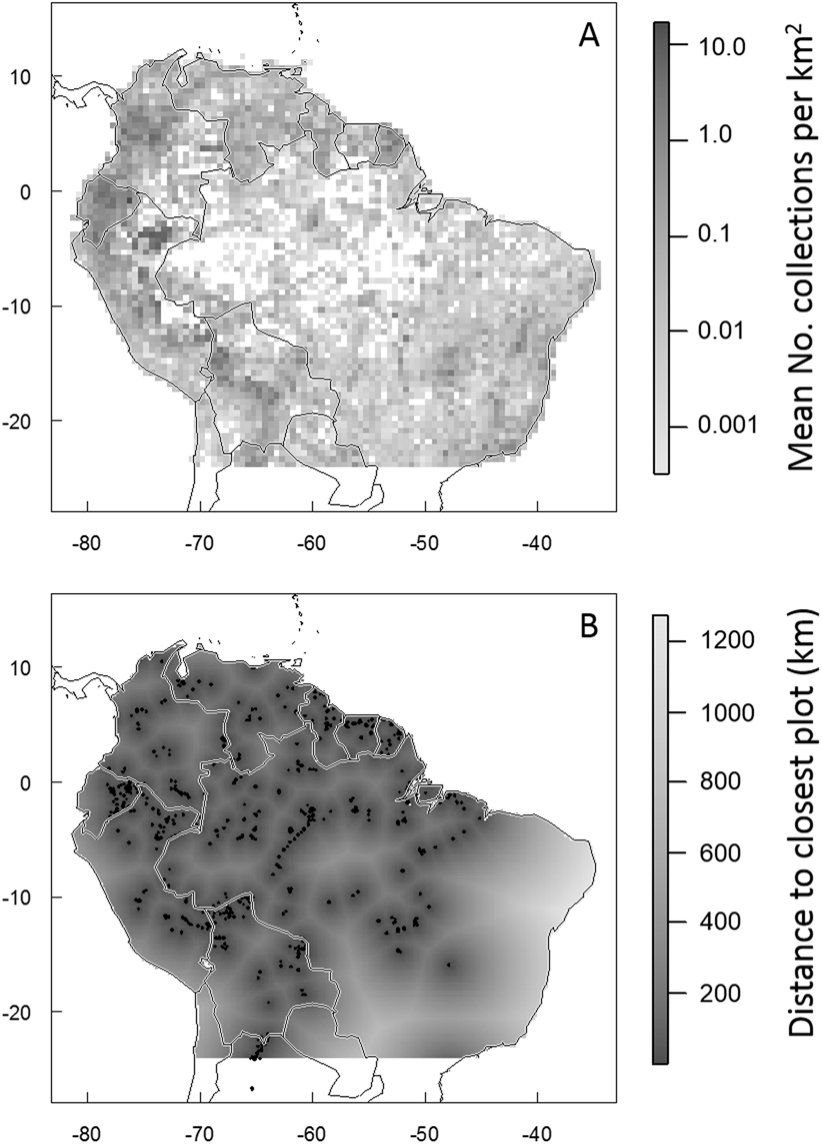
Density of occurrence records and distance to census plots in tropical South America. Panel A maps the mean density of plant occurrence records available in 2013 for tropical South America. Density is mapped at a spatial resolution of 0.5 x 0.5°, white pixels are areas where there are no available occurrence records. Panel B maps the distance from points in tropical South America to the closest vegetation census plot (mapped at a spatial resolution on 30 arc seconds).

**Fig 4:**
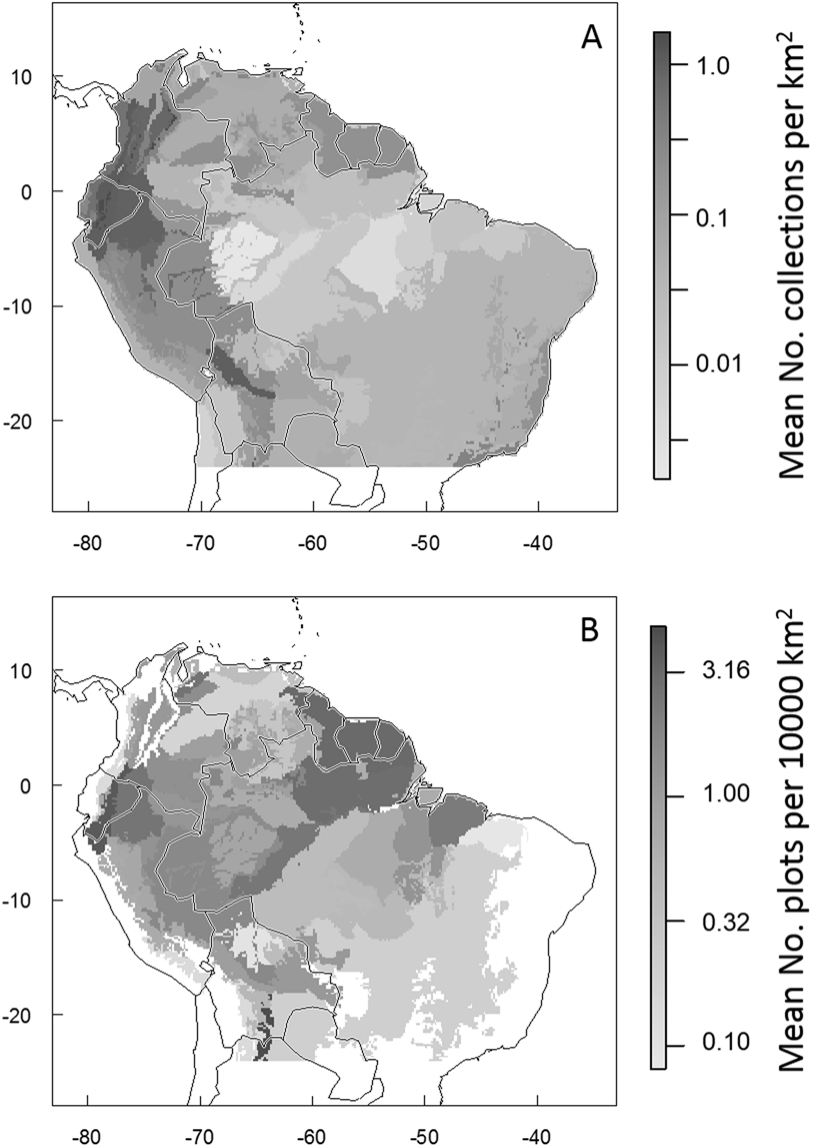
Density of occurrence records and census plots in ecoregions of tropical South America. Maps showing the mean density of (A) occurrence records and (B) vegetation census plots in each of tropical South American ecoregions.

In terms of spatial representation, the average and median density of collections has increased by an order of magnitude since 2007 (Table 1 and Fig 2b). In 2007, the majority of tropical South America was unrepresented by any GBIF herbarium collections (when collections are aggregated at a spatial scale of 0.5° latitude/longitude); by 2013, more than 85% of tropical South America was represented by at least one GBIF collection (Table 1 and Fig 3a). The density of collections varies greatly across space (Fig 3a) and between ecoregions (Table 2 and Fig 4a) and climatic zones (Tables 3 and 4). The greatest density of collections comes from the Northern Andean Paramo ecoregion. This ecoregion is relatively small (approximately 25000 km^2^) but is represented by more than 40000 unique plant collections (Table 2). The 2^nd^ through 5^th^ best collected ecoregions are also Andean (High Monte, Northwestern Andean Montane Forests, Eastern Cordillera Real Montane Forests, and Bolivian Yungas). Accordingly, the greatest density of collections come from cool, wet habitats such as those occurring in the montane Paramo. More generally, dryer areas and areas with hot (>20°C) or very cold (<10°C) mean annual temperatures are underrepresented in the GBIF database (Table 4).

**Table 2:** Density of collections data and census plots in different ecoregions of tropical South America (ecoregions defined according to the World Wildlife Fund; http://www.worldwildlife.org/publications/terrestrial-ecoregions-of-the-world; [23]).

Ecoregion name	Area (km^2^)*	No. collections	No. col / km^2^	No. plots	No. plots / 10000 km^2^
Cerrado	1914593	69234	0.036	39	0.204
Southwest Amazon moist forests	760847	196813	0.259	175	2.300
Caatinga	737482	27906	0.038	0	0.000
Madeira-Tapajos moist forests	721309	10059	0.014	33	0.458
Guianan moist forests	482415	97136	0.201	233	4.830
Uatuma-Trombetas moist forests	470092	10173	0.022	203	4.318
Mato Grosso seasonal forests	410995	14911	0.036	20	0.487
Llanos	380653	20805	0.055	6	0.158
Dry Chaco	347572	16802	0.048	7	0.201
Tapajos-Xingu moist forests	338782	1259	0.004	27	0.797
Alto Parana Atlantic forests	310138	10593	0.034	0	0.000
Japura-Solimoes-Negro moist forests	271443	3714	0.014	22	0.810
Xingu-Tocantins-Araguaia moist forests	267363	2276	0.009	45	1.683
Napo moist forests	258925	250810	0.969	96	3.708
Jurua-Purus moist forests	244283	431	0.002	25	1.023
Bahia interior forests	230463	12721	0.055	0	0.000
Chiquitano dry forests	230418	20922	0.091	35	1.519
Guianan piedmont and lowland moist forests	229187	13029	0.057	7	0.305
Central Andean dry puna	211583	3388	0.016	0	0.000
Negro-Branco moist forests	204934	43609	0.213	25	1.220
Caqueta moist forests	196159	7201	0.037	39	1.988
Tocantins/Pindare moist forests	194526	3441	0.018	65	3.341
Peruvian Yungas	186496	43757	0.235	16	0.858
Sechura desert	185253	8100	0.044	0	0.000
Solimoes-Japura moist forests	178847	30188	0.169	29	1.621
Purus-Madeira moist forests	174201	765	0.004	62	3.559
Pantanal	170444	3111	0.018	0	0.000
Central Andean puna	157648	16545	0.105	2	0.127
Purus varzea	150857	2929	0.019	32	2.121
Guianan Highlands moist forests	144118	15749	0.109	15	1.041
Maranhao Babatu forests	143813	1800	0.013	1	0.070
Beni savanna	123015	3492	0.028	1	0.081
Central Andean wet puna	122709	16060	0.131	0	0.000
Ucayali moist forests	116663	38358	0.329	11	0.943
Atlantic dry forests	112157	8017	0.071	0	0.000
Bahia coastal forests	112007	21895	0.195	0	0.000
Magdalena Valley montane forests	108222	90259	0.834	17	1.571
Eastern Cordillera real montane forests	105832	110215	1.041	67	6.331
Guianan savanna	105492	5861	0.056	8	0.758
Iquitos varzea	103580	66528	0.642	18	1.738
Rio Negro campinarana	94963	2722	0.029	24	2.527
Bolivian Yungas	92087	95328	1.035	12	1.303
Marajo varzea	87750	527	0.006	7	0.798
Northwestern Andean montane forests	83136	91144	1.096	1	0.120
Atacama desert	81276	529	0.007	0	0.000
Magdalena-Uraba moist forests	76294	6196	0.081	0	0.000
Bolivian montane dry forests	73599	17495	0.238	4	0.543
Cordillera Oriental montane forests	69768	22947	0.329	0	0.000
Monte Alegre varzea	69280	654	0.009	24	3.464
La Costa xeric shrublands	68871	4332	0.063	3	0.436
Apure-Villavicencio dry forests	68637	6859	0.100	14	2.040
Choco-Darian moist forests	60304	36148	0.599	0	0.000
Humid Chaco	57333	3395	0.059	0	0.000
Serra do Mar coastal forests	55436	19440	0.351	0	0.000
Pantepui	48698	8866	0.182	3	0.616
Southern Andean Yungas	44794	11735	0.262	35	7.813
Tumbes-Piura dry forests	42161	1977	0.047	0	0.000
Cauca Valley montane forests	34628	30424	0.879	4	1.155
Western Ecuador moist forests	33726	22367	0.663	0	0.000
Amazon-Orinoco-Southern Caribbean mangroves	33167	5505	0.166	2	0.603
Maracaibo dry forests	30841	1485	0.048	2	0.648
Venezuelan Andes montane forests	29935	8765	0.293	12	4.009
Guajira-Barranquilla xeric scrub	28053	2168	0.077	4	1.426
Orinoco Delta swamp forests	27895	1227	0.044	1	0.358
Campos Rupestres montane savanna	26940	8806	0.327	0	0.000
Sin· Valley dry forests	26434	2133	0.081	0	0.000
Northern Andean paramo	24388	40023	1.641	2	0.820
Ecuadorian dry forests	22366	6670	0.298	0	0.000
Pernambuco interior forests	21750	1138	0.052	0	0.000
Catatumbo moist forests	21075	1490	0.071	0	0.000
Magdalena Valley dry forests	19231	7324	0.381	0	0.000
Pernambuco coastal forests	17689	1343	0.076	0	0.000
Lara-Falcon dry forests	17577	935	0.053	0	0.000
Paraguana xeric scrub	15553	204	0.013	0	0.000
Cordillera La Costa montane forests	15243	4418	0.290	0	0.000
Maranon dry forests	12314	3797	0.308	0	0.000
Cordillera Central paramo	11595	1500	0.129	0	0.000
Northeastern Brazil restingas	11002	81	0.007	0	0.000
Gurupa varzea	9633	107	0.011	0	0.000
Araucaria moist forests	7556	309	0.041	0	0.000
Guianan freshwater swamp forests	6850	90	0.013	0	0.000
South American Pacific mangroves	6534	1405	0.215	0	0.000
Araya and Paria xeric scrub	6090	482	0.079	0	0.000
Southern Atlantic mangroves	6019	918	0.153	0	0.000
Orinoco wetlands	5432	21	0.004	0	0.000
Santa Marta montane forests	5072	2568	0.506	0	0.000
Cauca Valley dry forests	4803	3138	0.653	0	0.000
Atlantic Coast restingas	3299	424	0.129	0	0.000
Guayaquil flooded grasslands	3096	1263	0.408	0	0.000
Caatinga Enclaves moist forests	3089	98	0.032	0	0.000
Cordillera de Merida paramo	2721	827	0.304	0	0.000
Chilean matorral	2211	18	0.008	0	0.000
Patia Valley dry forests	2064	97	0.047	0	0.000
Santa Marta paramo	1352	378	0.280	0	0.000
High Monte	1261	1397	1.108	0	0.000
Eastern Panamanian montane forests	341	26	0.076	0	0.000

* area only within tropical South America; some ecoregions may extend beyond this region.

**Table 3:** The extent of land area (km2) under different climatic conditions as defined by current Total Annual Precipitation (TAP) and Mean Annual Temperature (MAT).

TAP (mm) MAT (°C)	0–500	500–1000	1000–1500	1500–2000	2000–3000	3000–4000	4000–6000	>6000	>0
**0–2**	7781	15654	0	0	0	0	0	0	23435
**2–4**	36708	54744	0	0	342	0	0	0	91452
**4–6**	49923	63869	3383	342	1019	0	0	0	118536
**6–8**	75382	73829	7499	2400	1717	0	0	0	160827
**8–10**	104778	65755	13303	6523	1019	0	0	0	191378
**10–12**	44748	35271	23235	8590	2734	0	0	0	114578
**12–14**	44012	46770	19396	7882	4451	0	0	0	122511
**14–16**	51591	43582	23777	19147	11294	342	0	0	149732
**16–18**	53790	45071	29256	32344	25799	3776	0	0	190035
**18–20**	46792	42818	123692	82946	28958	9591	677	0	335475
**20–22**	11238	162000	365341	119160	72751	27076	4109	0	761676
**22–24**	85953	460572	782311	466536	126930	43135	12599	685	1978721
**24–26**	52874	550604	800039	1322593	1805551	420056	22959	13373	4988049
**26–28**	5399	201323	500020	1038270	2339775	220335	9575	14750	4329448
**28–30**	5056	9459	15610	9520	35546	3409	1705	0	80305
**>0**	676025	1871321	2706862	3116252	4457545	727720	51625	28808	13636159

**Table 4:** The density of collection records (No. col / km2) available for areas under different climatic conditions as defined by current Total Annual Precipitation (TAP) and Mean Annual Temperature (MAT).

TAP (mm) MAT (°C)	0–500	500–1000	1000–1500	1500–2000	2000–3000	3000–4000	4000–6000	>6000	>0
**0–2**	0.018	0.014	-	-	-	-	-	-	0.009
**2–4**	0.010	0.053	-	-	6.376	-	-	-	0.032
**4–6**	0.019	0.115	0.844	0.056	3.553	-	-	-	0.117
**6–8**	0.069	0.162	1.440	0.767	0.474	-	-	-	0.158
**8–10**	0.078	0.205	0.890	1.586	0.692	-	-	-	0.190
**10–12**	0.045	0.642	1.334	0.847	0.520	-	-	-	0.544
**12–14**	0.079	0.513	0.859	0.740	1.240	-	-	-	0.425
**14–16**	0.022	0.481	0.791	0.628	0.898	0.056	-	-	0.414
**16–18**	0.022	0.349	1.007	0.450	0.762	0.753	-	-	0.433
**18–20**	0.063	0.446	0.271	0.336	0.602	0.851	0.095	-	0.317
**20–22**	0.022	0.162	0.121	0.256	0.430	0.457	1.875	-	0.200
**22–24**	0.029	0.048	0.067	0.067	0.183	0.541	0.966	0.026	0.083
**24–26**	0.056	0.030	0.049	0.069	0.125	0.365	0.186	0.803	0.109
**26–28**	0.009	0.012	0.030	0.091	0.131	0.158	0.420	1.181	0.110
**28–30**	0.003	0.025	0.025	0.052	0.096	0.102	0.056	-	0.062
**>0**	0.046	0.110	0.113	0.105	0.146	0.323	0.549	0.978	0.131

Combined, the included plot networks represent 1535 census plots distributed throughout tropical South America (Table 2 and Fig 1h). Any given point in tropical South America is an average of 235.7 km from the closest census plot (median distance = 149.2 km; Fig 3b). 1.3% of tropical South America is within 10 km of the closet census plot; 14.9% is within 50 km of a plot; 33.8% is within 100 km of a plot; and 86.7% is within 500 km of a plot. The greatest distance from any point in tropical South America to its closest of the included plots is 1273.3 km. As with the collections data, the greatest concentration of plots are in the tropical montane ecoregions (the Southern Andean Yungas, Eastern Cordillera Real Montane Forests, and Venezuelan Andes Montane Forests are the best, second-best, and fifth-best represented ecoregions, respectively; Table 2 and Fig 4b). The best-represented climatic zones, by far, are the areas with 4000–6000 mm rainfall and mean annual temperatures of 20–22°C, and areas with 3000–4000 mm rainfall and mean annual temperatures of 16–18°C. The other climatic zones have markedly lower densities of plots (Table 5).

**Table 5:** The density of census plots (No. plots / 10000 km2) in areas under different climatic conditions as defined by current Total Annual Precipitation (TAP) and Mean Annual Temperature (MAT).

TAP (mm) MAT (°C)	0–500	500–1000	1000–1500	1500–2000	2000–3000	3000–4000	4000–6000	>6000	>0
**0–2**	0.000	0.000	-	-	-	-	-	-	0.000
**2–4**	0.000	0.000	-	-	0.000	-	-	-	0.000
**4–6**	0.000	0.000	0.000	0.000	0.000	-	-	-	0.000
**6–8**	0.000	0.000	2.667	0.000	0.000	-	-	-	0.124
**8–10**	0.000	0.000	0.000	0.000	0.000	-	-	-	0.000
**10–12**	0.000	0.567	0.000	2.328	0.000	-	-	-	0.349
**12–14**	0.454	0.000	0.000	17.762	4.494	-	-	-	1.469
**14–16**	0.000	1.147	0.841	0.000	1.771	0.000	-	-	0.601
**16–18**	0.000	1.331	6.153	0.000	2.713	42.375	-	-	2.473
**18–20**	0.000	1.868	0.081	0.241	0.000	8.341	0.000	-	0.566
**20–22**	0.000	0.370	0.849	0.755	0.825	0.369	55.968	-	0.998
**22–24**	0.000	0.000	0.179	0.514	0.551	2.550	0.794	0.000	0.288
**24–26**	0.000	0.054	0.525	0.832	1.313	2.643	0.000	0.000	1.008
**26–28**	0.000	0.248	0.220	0.905	2.740	2.088	0.000	0.000	1.841
**28–30**	0.000	0.000	0.000	1.050	0.563	0.000	0.000	-	0.374
**>0**	0.030	0.187	0.447	0.821	2.028	2.652	4.649	0.000	1.126

## Discussion

In order to advance conservation science we need to overcome the Wallacean and Linnean shortfalls [29–31]. In other words, we need to know what species are out there and where they occur [32]. One tool that can help us to bypass these shortfalls is the rapidly expanding availability of natural history and collections data. For tropical South America, the amount of collections data that is available online has skyrocketed over the past 2 decades (Fig 1a–1g). Indeed, since the launch of GBIF in 2007, the number of records available from tropical South America has increased by nearly 60% annually.

The rapid increase in available collection data has led to a marked decrease in the “data void”; however, the data void still exists and in some regards remains unacceptably large. This is because the majority of newly-added collections have gone towards increasing the number of species represented but relatively few collections go towards augmenting the sample size of records available for the already-collected species. In other words, the records now include many more species than previously. For example, the number of species represented in the available data rose from <15,000 species in 2007 to >52,000 species in 2013. However, most species remain so poorly represented that they are functionally invisible to ecological studies since they have too small of sample sizes to be included in most modelling exercises or conservation assessments (e.g., in 2013, <14,000 species [26%] had 20 or more available records while >30,000 species [57%] are represented by fewer than 10 records and nearly 10,000 species [19%] are represented by just a single record; Fig 2a). It is also highly likely that there are many more species that remain unnamed or that are not represented by the online databases [31,33]. Indeed, many species will probably never be represented in herbaria or online databases due to their rarity as well as the difficulty of collecting flowers and fruits which are often needed to accurately identify the collections to species. The vast majority of specimens gathered for ecological studies in the tropics are sterile; therefore many collections are not identified to species or are identified incorrectly [6].

Likewise, from a spatial perspective, the data void has shrunk but still remains distressingly large. To date, more than 10% of tropical South America is still represented by no collection records at all and an additional 15% remains with a density of less than 0.0005 available records per km^2^—or in other words, with just one collection for every 2000 km^2^ (Fig 3a). Indeed, the overall collection density across the region is approximately 1 collection record for every 10 km^2^. Making matters worse, the density of collection records is not evenly distributed amongst habitat types or climatic zones. Many ecoregions are very poorly represented in the GBIF collections database. For example, the Cerrado is one of the South America’s largest, most diverse, and most threatened ecoregions [4,34] but it is represented by an average of just one record for approximately every 30 km^2^. The Caatinga Dry Forest ecoregion of northern Brazil is represented by an approximately equal density of collection records while other ecoregions such as the Madeira-Tapajos Moist Forests are represented by even lower densities of records (1 collection for every 70 km^2^). In contrast, several of the Andean ecoregions are relatively well-represented with densities of more than 1 collection record per km^2^ (Table 2). In accord with these patterns, there are also large disparities between climatic zones in their collection intensities. Hot, dry habitats are the least-represented, potentially due to lower diversities and densities of plants in these area. However, there are other very large and relatively-hospitable climate zones which are also very poorly represented. For example, large parts of tropical South America have mean annual rainfalls of between 1500–2000mm and mean annual temperatures of 24–28°C, but these areas are not well-represented in the collections database (Table 4). This may be due to a lack of access (due to physical or bureaucratic impediments) or infrastructure in these areas.

Natural history and herbarium records are only one form of data. In the past decades there have been multiple independent efforts to collate and standardize census data. However, even when combining these efforts the number of plots pales in comparison to the extent and diversity of tropical South America. Across all of the included networks, there are a total of approximately 1500 plots (Fig 1h) covering roughly 15 km^2^ of forest in tropical South America. In other words, combining all our efforts we are still only censusing about 0.0001% of the total land area (or <0.0003% of the Amazon). For comparison, this is 1/20^th^ the spatial density of plots in the USA that are maintained by just the US Forest Services’ Forest Inventory and Analysis Program alone (http://fia.fs.fed.us/).

Most places in tropical South America are more than 150 km from the closest census plot (Fig 3b) and most ecoregions in tropical South America have less than 1 plot per 30,000 square km^2^ (Fig 4b). The relative density of census plots across South American ecoregions is not significantly correlated with the density of collection records (Pearson correlation coefficient R = 0.16). In other words, habitats that are well represented in the plot networks are often only poorly-represented in the collections data and vice versa (Table 2). In some cases, this discrepancy is understandable given the emphasis of the included plot networks on tree census and hence the preclusion of plots from some areas with low forest cover but with high plant diversities and thus many collections (such as the Northern Andean Paramo ecoregion). As such, it is possible that the discrepancy between plot and collection densities could be reduced through the inclusion of additional plots focused on non-forest habitats (for example, the GLobal Observation Research Initiative in Alpine Environments network [GLORIA; http://www.gloria.ac.at/] promotes standardized methodologies to census alpine vegetation around the world, including in the high Andes; the GLORIA network was not included in this analysis because their plot locations and data are not readily available online). In other cases (e.g., high plot density but low collection density in the Monte Alegre Varzea and Southern Andean Yungas ecoregions, and vice versa in the Western Ecuador Moist Forests ecoregion), the discrepancy in plot vs. collection density has no apparent explanation other than differential sampling efforts between habitats and regions. Interestingly, the density of neither plots nor collections appears to be strongly linked to the actual representation of the different habitats in ecological studies. For example, Andean ecosystems are greatly underrepresented in the ecological literature but are fairly well-represented in the two types of databases explored here [35]. Given the high spatial variability in composition, structure and dynamic across the Amazon and Tropical South America, there is a clear need for more and better-distributed census plots, collection campaigns, and research efforts.

From a diversity standpoint, it is hard to assess the representativeness of the census plots. This is because many species remain unidentified [31] and nomenclature and taxonomy has still not been fully standardized between, or even within, separate plot networks [36]. In one recent study [6], the taxonomy and nomenclature of collections from the ATDN, the largest of the tropical plot networks, was standardized. The ATDN’s network of 1170 plots were found to include 4,962 species of trees. This is only 30% of the estimated Amazonian tree diversity and less than 10% of the total tropical South American plant diversity as represented in the herbarium collection database. Furthermore, most species were poorly-represented within and across the plots. 21% of represented species occurred within just a single plot and 13% had only a single individual [6]. In other words, as with the herbarium records, most species remain functionally invisible to ecology and conservation studies due to small sample sizes. Making matters worse, census plot data are not usually open access. Each plot network understandably has its own policies for data distribution and sharing, but in most cases data are provided only upon request for use in specific, pre-approved analyses. In other words, census data are not typically available for data mining, data exploration, preliminary studies, or for general assessments (for this study I only used the published locations of plots and not any actual plot data). Limited access to data can hinder the advancement of large-scale ecological, biogeographic and conservation studies; data should made publically available whenever possible (with proper attribution to the data creators and managers).

The results of this assessment clearly illustrate that while recent expansions of collection databases and census plot networks have greatly increased the amount of data available for tropical South America, the data void remains far from filled. There are many species that are still not adequately represented in either the collection and/or plot databases. There are also huge parts of tropical South America, containing many distinct and diverse habitats and climatic zones, that remain very poorly-represented in either the plot and/or collections databases. The lack of data for particular species, habitats and climate zones limits our ability to predict the impacts of climate change and other large-scale anthropogenic disturbances—especially when these disturbances themselves may disproportionately impact different species and habitats. This limitation is expected to be even more severe in other tropical regions, such as Africa and Southeast Asia, where data availability is generally lower [22].

To conclude, I stress that my goal in this study is not to criticize the workers who are diligently adding to or administrating the natural history databases and plot networks. Quite the opposite, my motivation is to help highlight how valuable these data are, and how important ongoing efforts at increasing sample sizes through the generation of new data and the publishing of existing datasets will be [18,37]. Simply put, we need more data.

## Supporting Information

S1 File Contributing Herbaria. Citations for herbaria and databases contributing information to the Global Biodiversity Information Facility as used in this study (Data was downloaded from GBIF in January and February 2014).
(DOCX)
