# Supplementary material for: Are We Filling the Data Void? An Assessment of the Amount and Extent of Plant Collection Records and Census Data Available for Tropical South America
Source: PLoS One. 2015 Apr 30;10(4):e0125629. doi: 10.1371/journal.pone.0125629 (PMC4416035; doi:10.1371/journal.pone.0125629)
Supplement: S1 File — Citations for herbaria and databases contributing information to the Global Biodiversity Information Facility as used in this study (Data was downloaded from GBIF in January and February 2014). (DOCX) [file pone.0125629.s001.docx]

**Supplemental Online Material:**

Citations for herbaria and databases contributing information to the Global Biodiversity Information Facility as used in this study (Data was downloaded from GBIF in January and February 2014).

Levy Tacher, S. I. 1999. Contribución al conocimiento de la flora útil de la selva Lacandona. Conservation International México A.C. Bases de datos SNIB2010-CONABIO. Proyecto No. M002. México, D.F.

Valdés Reyna, J. y P. Dávila Aranda. 1998. Base de datos de las gramíneas (Poaceae) del noreste de México. Universidad Autónoma Agraria Antonio Narro. Bases de datos SNIB2010-CONABIO proyecto No. G029. México, D.F.

Berkeley Natural History Museums: UCBG TAPIR Provider

Museo de La Salle - MLS (2011 - ). Herbario Museo de La Salle Bogotá (MLS), 11371 Registros, aportados por Espitia-Barrera JE (Publicador, Creador del Recurso, Proveedor de los metadatos), Aponte-Rojas AM (Procesador), En línea, http://evirtual.lasalle.edu.co:8080/ipt/, Versión 1.0 (última modificación en 03/12/2012), http://evirtual.lasalle.edu.co:8080/ipt/

Colección de Diatomeas del Sur del Golfo de México (ICMyL-DF, UNAM)

Téllez Valdés, O. y J. Martínez. 2000. Base de datos de la flora de la Reserva de la Biosfera Chamela-Cuixmala, Jalisco, México. Universidad Nacional Autónoma de México. Instituto de Biología. Bases de datos SNIB2010-CONABIO proyecto No. L289. México, D.F.

EURISCO, the European PGR Search Catalogue http://eurisco.ecpgr.org/_1About/9Legal/index.php

Herbario XAL del Instituto de Ecología, A.C., México (IE-XAL)

MEXUPVT, Portal UNIBIO, Instituto de Biología, Universidad Nacional Autónoma de México, http://www.unibio.unam.mx consultada el dd/mm/yy.

Administración de Parques Nacionales, Argentina: Registros de hongos en áreas protegidas de la Argentina

Rigby,P.R., B.Konar, T.Kato, K.Iken, H.Chenelot and Y.Shirayama (2005)NaGISA OBIS Dataset ver.1

University of British Columbia Herbarium (UBC). http://www.biodiversity.ubc.ca/museum/herbarium/database.html. (consulted on [date]), http://www.biodiversity.ubc.ca/museum/herbarium/database.html

Botanical Institute of Barcelona (CSIC - Ayuntamiento de Barcelona): Institut Botanic de Barcelona, BC-Histórico

University of Kansas Biodiversity Institute: Botany Vascular Plant Collection

New Mexico Biodiversity Collections Consortium internet accessible database, provided through DiGIR protocol.

Missouri Botanical Garden: Missouri Botanical Garden

Riemann González, H. 1999. Riqueza y distribución de especies vegetales en la Península de Baja California. El Colegio de la Frontera Norte A.C. Bases de datos SNIB2010-CONABIO proyecto No. H016. México, D.F.

MEXULEGUMINOSAE, Portal UNIBIO, Instituto de Biología, Universidad Nacional Autónoma de México, http://www.unibio.unam.mx consultada el dd/mm/yy.

Eguiarte Fruns, L. E. y G. R. Furnier. 1997. Niveles y patrones de variación genética del género Abies en México. Universidad Nacional Autónoma de México. Instituto de Ecología. Bases de datos SNIB2010-CONABIO proyecto No. B138. México, D.F.

Musée national d'histoire naturelle Luxembourg: Biological and palaeontological collection and observation data MNHNL

Sturm H., O. Rangel. 1985. Ecologia de los Paramos Andinos: una visión preliminar integrada. Instituto de Ciencias Naturales - Universidad Nacional de Colombia. Bogota. 292p., n/a

Siqueiros Beltrones, D. A. 1999. Estructura y variación geográfica de las asociaciones de diatomeas bentónicas de la Península de Baja California; Bahía de La Paz. Universidad Autónoma de Baja California Sur. Bases de datos SNIB2010-CONABIO proyecto No. H031. México D. F.

Corporación Autónoma Regional del Centro de Antioquia (2009 - ). Inventario de fauna presente en la jurisdicción de Corantioquia, 1668 Registros, aportados por Arango AM( Publicador, Creador del Recurso, Proveedor de los Metadatos), Rodríguez W (Autor), Callejas LF (Autor), Callejas-Posada R (Autor), Colorado-López (Autor), En línea, http://ipt.sibcolombia.net/sib/resource.do?r=corantioquia-2008-helechos, Versión 1 (última modificación el 01/12/2012)., http://ipt.sibcolombia.net/sib/resource.do?r=corantioquia-2008-helechos

González González, J. 2002. Inventario de macroalgas de Bahía de Banderas: Fase I y Fase II. Universidad Nacional Autónoma de México. Facultad de Ciencias. Bases de datos SNIB2010-CONABIO proyecto No. S124. México, D.F.

National geological collection of Estonia: www.geocollections.info; University of Tartu, Museum of Geology

Department of Botany, Research and Collections Information System, NMNH, Smithsonian Institution. See: http://www.mnh.si.edu/rc/db/2data_access_policy.html

Colección de Monocotiledóneas Mexicanas (UAM-I)

Yale Peabody Museum, (c) 2009. Specimen data records available through distributed digital resources.

Botanical Institute of Barcelona (CSIC - Ayuntamiento de Barcelona): Institut Botanic de Barcelona, BC

Canadian Museum of Nature Herbarium (The National Herbarium of Canada)

Gutiérrez Garduño, M. V. 1999. Catálogos florísticos de México por entidad federativa e información etnobotánica de la Colección del Herbario Nacional Biól. Luciano Vela Gálvez (INIF). Secretaría de Agricultura, Ganadería y Desarrollo Rural. Instituto Nacional de Investigaciones Forestales Agrícolas y Pecuarias. Bases de datos SNIB2010-CONABIO proyecto No. J010. México, D.F.

Buitrón Sánchez, B E. 2005. Sistematización de la Colección Paleontológica de la Facultad de Ingeniería, UNAM. Universidad Nacional Autónoma de México. Facultad de Ingeniería. Bases de datos SNIB2010-CONABIO. Proyecto No. V045. México, D.F.

University of Connecticut: CONN

Royal Botanic Garden Edinburgh. (2013) Preserved Collections Database (E)

Renker, C. (Ed.) 2010+ (continuously updated): Digitised specimen data at Naturhistorisches Museum Mainz / Landessammlung für Naturkunde Rheinland-Pfalz (MNHM).

Harvard University Herbaria, Index of Botanical Specimens., Harvard University Herbaria, Index of Botanical Specimens

Instituto de Investigaciones Ambientales del Pacífico - IIAP (2013). Caracterización vegetal de una zona de alta montaña (litoral de San Juan) como herramienta de proyección para el establecimiento de una figura de conservación en el chocó biogeográfico 52 Registros, aportados por Ramirez-Moreno G (Publicador, Investigador principal), Valoyes-Cardozo Z (Creador del recurso, Proveedor de los metadatos), Klinger-Braham W (Autor), Mosquera-Benítez HD (Autor), En linea, http://ipt.sibcolombia.net/iiap/resource.do?r=flora_paramo_duende, publicado el 05/03/2013., http://ipt.sibcolombia.net/iiap/resource.do?r=flora_paramo_duende

Masure, Edwige (1988): (Figure 3) Distribution of dinoflagellate cysts in the mid-Cretaceous of ODP Hole 101-635B, doi:10.1594/PANGAEA.742969

Instituto de Ciencias Naturales: Instituto de Ciencias Naturales

Oleoducto Bicentenario (2013). RESCATE DE EPÍFITAS OLEODUCTO BICENTENARIO, TRAMO ARAGUANEY - BANADÍA (SIEMBRA) 944. Registros, aportados por Alejandro Calderón (Publicador, Proveedor de los Metadatos, Proveedor de Contenido, Creador del Recurso). En linea, http://ipt.sibcolombia.net/sib/resource.do?r=epifitas_siembra, publicado el 08/05/2013.

Villanueva Gutiérrez, R. Subproyecto Néctar: En: Pozo de la Tijera, M del C y S. Calmé. 2005. Uso y monitoreo de los recursos naturales en el Corredor Biológico Mesoamericano (áreas focales Xpujil-Zoh Laguna y Carrillo Puerto). El Colegio de la Frontera Sur. Unidad Chetumal. Bases de datos SNIB2010-CONABIO proyecto No.BJ002. México, D.F

Sosa Ortega, V. 1995. Recuento de la diversidad florística de Veracruz. Instituto de Ecología A.C. Bases de datos SNIB2010-CONABIO proyecto No. P011. México, D.F.

Fundación ALMA (2012). Registros restauración ecológica participativa de la biodiversidad de cuatro complejos de humedal en el Magdalena medio. 61 Registros, aportados por Gutierrez-Camargo JC (Publicador, Proveedor de los Metadatos, Proveedor de Contenido), Chizacá L (Procesador), Montoya N (Procesador), En línea, http://ipt.sibcolombia.net/sib/resource.do?r=almamagmed, publicado el 10/09/2012, http://ipt.sibcolombia.net/sib/resource.do?r=almamagmed

Fragoso González, C. E. 1997. Biodiversidad de Veracruz: Oligochaeta. Annelida. Instituto de Ecología AC. Bases de datos SNIB2010-CONABIO proyecto No. E009. México, D.F.

Mora Olivo, A. 2003. Flora vascular acuática de la cuenca del Río Tamesí. Universidad Autónoma de Tamaulipas. Instituto de Ecología Aplicada. Bases de datos SNIB2010-CONABIO proyecto No. S078. México, D.F.

Chaisson, William P; Sigurdsson, H; Leckie, R Mark; Acton, Gary D; Shipboard Scientific Party, (2005): Range table from planktonic foraminifers in Hole 165-999A, doi:10.1594/PANGAEA.315445

Boeckel, Babette (2003): Coccolith percentage exclusive Emiliania huxleyi of surface sediment samples, doi:10.1594/PANGAEA.131214

Yale Peabody Museum, (c) 2009. Specimen data records available through distributed digital resources.

Vega Aviña, R. 2000. Catálogo y base de datos preliminar de la flora de Sinaloa. Universidad Autónoma de Sinaloa. Facultad de Agronomía. Bases de datos SNIB2010-CONABIO proyecto No. L057. México, D.F.

Meave del Castillo, M. E. 2009. Dinoflagelados y Diatomeas del Pacífico tropical mexicano. Universidad Autónoma Metropolitana. Unidad Iztapalapa. Bases de datos SNIB2010-CONABIO proyectos No. DJ022, BA008, S151 y H176. México D. F.Meave del Castillo, M. E. 2009. Dinoflagelados y Diatomeas del Pacífico tropical mexicano. Universidad Autónoma Metropolitana. Unidad Iztapalapa. Bases de datos SNIB2010-CONABIO proyectos No. DJ022, BA008, S151 y H176. México D. F.

Rangel-Ch., O and Ariza N. C. 2000. La vegetacion paramuna de los volcanes de Nariño. In: Colombia Diversidad Biotica III, la región de Vida paramuna de Colombia. Rangel-Ch. J.O. 2000. Universidad Nacional de Colombia. Editorial Unibiblios. Bogotá, Colombia., ISBN 958-701-010-8

Instituto de Botánica Darwinion - CONICET: Instituto de Botánica Darwinion

Guadarrama Olivera, M. de los A. 2000. Flora de la reserva de la biósfera de los Pantanos de Centla, en el estado de Tabasco, México. Universidad Juárez Autónoma de Tabasco. Bases de datos SNIB2010-CONABIO. Proyecto No. L138. México, D.F.

Reygadas Prado, D. D. 1999. Sistema de apoyo a la toma de decisiones para la reforestación rural en México. Secretaría de Agricultura, Ganadería, Desarrollo Rural, Pesca y Alimentación. Instituto Nacional de Investigaciones Forestales Agrícolas y Pecuarias. Bases de datos SNIB2010-CONABIO proyecto No. J063. México, D.F.

Applegate, S. P. y M. del C. Perrilliat Montoya. 1999. Propuesta para rescatar y conservar la paleobiota de la Cantera Tlayúa, en Tepexí de Rodríguez, Puebla: Fase II. Universidad Nacional Autónoma de México. Instituto de Geología. Bases de datos SNIB2010-CONABIO proyectos No. J086 y E011. México, D.F.

Balleza Cadengo, J. de J. 2005. Base de datos del Herbario de la Unidad Académica de Agronomía de la Universidad Autónoma de Zacatecas. Universidad Autónoma de Zacatecas. Facultad de Agronomía. Bases de datos SNIB2010-CONABIO proyecto No. AC001 y L114. México, D.F.

Department of Plant Biology. Faculty of Biological Sciences. Univ. Murcia: Universidad de Murcia, Dpto. Biología Vegetal (Botánica), Murcia: MUB-HEPATICAE

Museo Nacional de Costa Rica: herbario

Herbario de la Universidad de Alicante, ABH (2005).

MEXUPV, Portal UNIBIO, Instituto de Biología, Universidad Nacional Autónoma de México, http://www.unibio.unam.mx consultada el dd/mm/yy.

Ríos Jara, E. y R. Ramírez Delgadillo, 2008. Inventario de la biota terrestre (Florístico) y marina (Invertebrados, peces y macroalgas bentónicos) del Parque Nacional Isla Isabel. Universidad de Guadalajara. Centro Universitario de Ciencias Biológicas y Agropecuarias. Bases de datos SNIB2010-CONABIO. Marina. Proyecto No. BK018. México. D. F.

Chaisson, William P; Keigwin, Lloyd D; Rio, Domenico; Acton, Gary D; Shipboard Scientific Party, (2005): Range table from planktonic foraminifers in Hole 172-1060A, doi:10.1594/PANGAEA.315514

University of Washington Burke Museum. WTU Herbarium Vascular Plant Collection. Seattle, Washington.

Herbarium of Université de Montpellier 2, Institut de Botanique: Herbarium specimens

All the help and manifold efforts by persons and organisations who inspired and promoted the ongoing project of the digitization of botanical specimens "DigiBotA" is herewith gratefully acknowledged.

Lavrado, H.P. & Ignacio, B.L. (eds.) 2006. Biodiversidade bentônica da costa central da Zona Econômica Exclusiva brasileira. Rio de Janeiro : Museu Nacional, 2006.(Série Livros; 18) 389 p. ISBN 85-7427-014-8

Herbarium de Geo. B. Hinton, México

Botanic Garden and Botanical Museum Berlin-Dahlem: Culture Collection of Microalgae and Zygnematophyceae Collection Hamburg (MZCH-SVCK)

Asociación Becarios de Casanare - ABC (2009). PLantas laguna El Tinije, 1102 Registros, aportados por Ramírez B( Publicador, Creador del Recurso, Proveedor de los Metadatos), Casas LF (Autor), En línea, http://ipt.sibcolombia.net/sib/resource.do?r=abc-2009-plantas, publicado el 04/12/2012., http://ipt.sibcolombia.net/sib/resource.do?r=abc-2009-plantas

Continuous Plankton Recorder (CPR) data from the Sir Alister Hardy Foundation for Ocean Science (SAHFOS). Available from http://iobis.org/

Chávez Ortiz, E. A. 2006. Distribución e inventario de algunas especies bentónicas (hexacorales, octocorales, esponjas, y especies misceláneas) en arrecifes del Caribe mexicano. Instituto Politécnico Nacional. Centro Interdisciplinario de Ciencias Marinas. Bases de datos SNIB2010-CONABIO proyecto No. AS018. México, D.F.

Estrada Castillón, A. E. 2007. Flora del Parque Nacional Cumbres de Monterrey, Nuevo León, México. Universidad Autónoma de Nuevo León. Facultad de Ciencias Forestales. Bases de datos SNIB2010-CONABIO proyecto No. BK036. México, D.F.

Sipman, H. 1986 - (continuously updated): LICHCOL - database of the lichen collection at the Herbarium Berolinense (B).

Flores Villela, O. 1994. Historia natural del parque ecológico estatal de Omiltemi, Chilpancingo, Guerrero, México. Universidad Nacional Autónoma de México. Facultad de Ciencias. Bases de datos SNIB2010-CONABIO proyecto No. A004. México, D.F.

California Academy of Sciences: CAS Botany (BOT)

Bioversity International: SINGER Coordinator

Acadia University: E. C. Smith Herbarium (ACAD)

Instituto Nacional de Biodiversidad (INBio), Costa Rica: Especímenes INBio

Fairchild Tropical Botanic Garden Virtual Herbarium

Peñalba Garmendia, M. C. y A. Búrquez Montijo. 1999. Flora polínica de las llanuras de Sonora, al sur de Hermosillo. Universidad Nacional Autónoma de México. Instituto de Ecología. Bases de datos SNIB2010-CONABIO proyecto No. H189. México, D.F.

Árboles y Arbustos Nativos para la Restauración Ecológica y Reforestación de México (IE-DF,UNAM)

Yale Peabody Museum, (c) 2009. Specimen data records available through distributed digital resources.

ASTERACEAE, Portal UNIBIO, Instituto de Biología, Universidad Nacional Autónoma de México, http://www.unibio.unam.mx consultada el dd/mm/yy.

Velázquez, J. A. y F. J. Romero-Malpica. 1998. Análisis de la heterogeneidad ambiental y conectividad de las áreas naturales del sur del Valle de México. Universidad Nacional Autónoma de México. Facultad de Ciencias. Bases de datos SNIB2010-CONABIO proyecto No. B144. México, D.F.

Wildlife Sightings - junponline (http://www.junponline.com)

García Cruz, C. J. 1999. Manual ilustrado de las orquídeas silvestres del estado de Morelos. Instituto Chinoin A.C. Bases de datos SNIB2010-CONABIO proyecto No. H043. México, D.F.

University of Amsterdam / IBED: University of Amsterdam (NL) - Páramo pollen reference collection

Pando, F. et al. (2003). MA Cryptogamic collections online databases. http://www.rjb.csic.es/herbario/crypto/crydb.htm. (date when consulted)

Caluff, M., Serguera, M., Sánchez, C., Morejón, R., Regalado, L., Hernández, A. et Daniel A. 2006. Pteridophyte collection online database.

Administración de Parques Nacionales, Argentina: Plan de vertebrados de la Patagonia

Torre Cosío, J. y L. Bourillón Moreno. 2000. Inventario y monitoreo del Canal de Infiernillo para el comanejo de los recursos marinos en el territorio Seri, Golfo de California. Conservation International México A.C. Bases de datos SNIB2010-CONABIO proyecto No. L179. México, D.F.

Karl Franzens University of Graz, Insitute for Botany - Herbarium GZU: Herbarium GZU

MEXUBR, Portal UNIBIO, Instituto de Biología, Universidad Nacional Autónoma de México, http://www.unibio.unam.mx consultada el dd/mm/yy.

Robledo Ramírez, D. 1997. Conocimiento de la macroflora marina de interés económico de las Costas de Yucatán. Instituto Politécnico Nacional. Centro de Investigación y de Estudios Avanzados-Mérida. Bases de datos SNIB2010-CONABIO proyecto No. B077. México, D.F.

Watkins, David K; Verbeek, Joost W (1988): (Table 3) Nannofossil occurrence in the Albian of ODP Hole 101-635B, doi:10.1594/PANGAEA.743043

http://www.hmapcoml.org/Default.asp?ID=37

Instituto de Investigación de Recursos Biológicos Alexander von Humboldt. 2012. Ejemplares del herbario Federico Medem Bogotá - FMB, del Instituto Alexander von Humboldt. Instituto de Investigación de Recursos Biológicos Alexander von Humboldt, Villa de Leyva, Colombia, http://ipt.humboldt.org.co/resource.do?r=herbario_instituto_humboldt

Terrazas Salgado, T. 2000. Filogenia de las cactáceas columnares (Pachycereeae) con base en caracteres anatómico-morfológicos. Colegio de Postgraduados. Instituto de Recursos Naturales. Bases de datos SNIB2010-CONABIO proyecto No. L074. México, D.F.

Berkeley Natural History Museums: UCJEPS TAPIR Provider

Senckenberg: Herbarium Senckenbergianum (FR)

Instituto de Investigação Científica Tropical: IICT Herbário LISC

Centre for Genetic Resources, The Netherlands: Centre for Genetic Resources, the Netherlands, PGR passport data

Herrera Arrieta, Y. 2001. Manual de las gramíneas de Durango. Instituto Politécnico Nacional. Centro Interdisciplinario de Investigación para el Desarrollo Integral Regional-Durango. Bases de datos SNIB2010-CONABIO proyecto No. R035. México, D.F.

Sturm H., O. Rangel. 1985. Ecologia de los Paramos Andinos: una visión preliminar integrada. Instituto de Ciencias Naturales - Universidad Nacional de Colombia. Bogota. 292p., n/a

Espinosa Organista, D. 2006. Taxonomía y prospección del hábitat de las poblaciones de Bursera sect. Bullockia con especial énfasis en las especies afines al 'linaloe', B. aloexylon (Schiede ex Schlecht.) Engl. Universidad Nacional Autónoma de México. Facultad de Estudios Superiores Zaragoza. Bases de datos SNIB2010-CONABIO. Proyecto No. BS001. México, D.F.

MEXUPA, Portal UNIBIO, Instituto de Biología, Universidad Nacional Autónoma de México, http://www.unibio.unam.mx consultada el dd/mm/yy.

Herbario de la Universidad de Salamanca (SALA)

National Institute of Genetics, ROIS: AIS Wildtype Populations of Arabidopsis

GBIF-Sweden: Botany (UPS)

Herbario del CIBNOR

Skovmand, B. 1997. Colección, preservación y caracterización de cultivares criollos de origen español de trigo y centeno de México. Centro Internacional de Mejoramiento de Maíz y Trigo. Bases de datos SNIB2010-CONABIO proyecto No. E001. México, D.F.

University of Washington Burke Museum. WTU Herbarium Bryophyte Collection. Seattle, Washington.

Stocks, K. 2003. SeamountsOnline: an online information system for seamount biology. Version 3.1. seamounts.sdsc.edu

Martínez, M. 1999. Flora acuática de Querétaro. Universidad Autónoma de Querétaro. Facultad de Ciencias Naturales. Bases de datos SNIB2010-CONABIO proyecto No. H076. México, D.F.

Boersma, Anne; Perch-Nielsen, Katharina; Supko, Peter R (2005): Foraminifera abundance of Hole 39-357, doi:10.1594/PANGAEA.240087

Field Museum: Field Museum of Natural History (Botany) Seed Plant Collection

De la Torre Almaráz, R. 2001. Inventario fitopatológico de las especies vegetales dominantes en la región de Zapotitlán de las Salinas, Pue. Universidad Nacional Autónoma de México. Facultad de Estudios Superiores Iztacala. Bases de datos SNIB2010-CONABIO proyecto No. R013. México, D.F.

Luna Vega, M. I. 1999. Florística y biogeografía de algunos bosques mesófilos de la Huasteca Hidalguense: Fase II (Tlahuelompa y Eloxochitlán). Universidad Nacional Autónoma de México. Facultad de Ciencias. Bases de datos SNIB2010-CONABIO. Proyecto No. H102. México, D.F.

University of British Columbia Herbarium (UBC). http://www.biodiversity.ubc.ca/museum/herbarium/database.html. (consulted on [date]), http://www.biodiversity.ubc.ca/museum/herbarium/database.html

Tidal Creek Database, NOAA Oceans and Human Health Initiative, NOAA Hollings Marine Laboratory

Wolf, J. H. D. 2000. Ecología y biogeografía de epífitas vasculares de Chiapas, México. El Colegio de la Frontera Sur. Bases de datos SNIB2010-CONABIO proyectos No. L050 y B060. México, D.F.

Green Plant Herbarium (TRT) from Royal Ontario Museum. http://dx.doi.org/10.5886/g7j6gct1 (accessed on [date])., doi:10.5886/g7j6gct1

Mountain Invasion Research Network MIREN_ETH. Mountain Invasion Research Network (MIREN) survey

Pando, F. et al. (2003). MA Cryptogamic collections online databases. http://www.rjb.csic.es/herbario/crypto/crydb.htm. (date when consulted)

Bravo Marentes, C. 1999. Inventario nacional de especies vegetales y animales de uso artesanal. Asociación Mexicana de Arte y Cultura Popular A. C. Bases de datos SNIB2010-CONABIO proyecto No. J002. México, D.F.

MNHN - Museum national d'Histoire naturelle: Phanerogams herbarium specimens

Rangel-Ch., O and Ariza N. C. 2000. La vegetacion del Parque Nacional Natural Chingaza in Colombia Diversidad Biotica III, la región de Vida paramuna de Colombia. Rangel-Ch. J.O. 2000. Universidad Nacional de Colombia. Editorial Unibiblios. Bogotá, Colombia., ISBN 958-701-010-8

Colorado State University Herbarium (CSU): Colorado State University Herbarium

Rodríguez Contreras, A. 1999. Estudio sistemático y ecológico del género Tigridia (Iridaceae). Universidad de Guadalajara. Centro Universitario de Ciencias Biológicas y Agropecuarias. Bases de datos SNIB2010-CONABIO proyecto No. J089. México, D.F.

Biebow, Nicole (2003): Assemblage of dinoflagellate cysts analysed in sediment core SO78-159-1, doi:10.1594/PANGAEA.126415

Martínez y Pérez, J. L. 1999. Flora genérica de las gramíneas del estado de Tlaxcala. Universidad Autónoma de Tlaxcala. Centro de Investigación en Ciencias Biológicas. Bases de datos SNIB2010-CONABIO proyecto No. H148. México, D.F.

Biologiezentrum Linz Oberoesterreich: Biologiezentrum Linz

Equihua, C. 1999. Brioflora de la Reserva de Montes Azules, Chis.: Diversidad, biogeografía y depauperación por actividad humana. Universidad Nacional Autónoma de México. Instituto de Ecología. Bases de datos SNIB2010-CONABIO proyecto No. H285. México, D.F.

Vester, H. F. M. Subproyecto Bosques, En: Pozo de la Tijera, M del C y S. Calmé. 2005. Uso y monitoreo de los recursos naturales en el Corredor Biológico Mesoamericano (áreas focales Xpujil-Zoh Laguna y Carrillo Puerto). El Colegio de la Frontera Sur. Unidad Chetumal. Bases de datos SNIB2010-CONABIO proyecto No.BJ002. México, D.F.

University of Alabama Herbarium (UNA)

Repatriación de datos del Herbario de Arizona (ARIZ)

González Elizondo, M. 1999. Florística de áreas protegidas en el estado de Durango. Instituto Politécnico Nacional. Centro Interdisciplinario de Investigación para el Desarrollo Integral Regional-Durango. Bases de datos SNIB2010-CONABIO proyecto No. H100. México, D.F.

Jardin Botanique de Montréal (JBM) from Jardin Botanique de Montréal. http://dx.doi.org/10.5886/uxx7hzy3. (accessed on [date]), doi:10.5886/uxx7hzy3

Burckhardt D. Swiss Psyllid (Hemiptera) Collections - Basel. Naturhistorisches Museum Basel

Ceballos, G. 1997. Diversidad biológica y conservación del ecosistema de los perros de la pradera (Cymomys ludovicianus) en México. Universidad Nacional Autónoma de México. Instituto de Ecología. Bases de datos SNIB2010-CONABIO proyecto No. B043. México, D.F.

Magaña Cota, G. E. 2004. Colección científica del Museo de Historia Natural Alfredo Dugès. Universidad de Guanajuato. Bases de datos SNIB2010-CONABIO. Proyecto No. V002. México, D.F.

Ghana Biodiversity Information Facility (GhaBIF): University of Ghana - Ghana Herbarium

Department of Bioscience, Aarhus University: Aarhus University Palm Transect Database

M.E.Conkright, J.I. Antonov, O. Baranova, T.P. Boyer, H.E. Garcia, R. Gelfeld, D. Johnson, R.A. Locarnini, P.P., T.D. O'Brien, I. Smolyar, C. Stephens, 2002: World Pcean Database 2001, Voulme 1: Introduction. Ed: Sydney Levitus, NOAA Atlas NESDIS 42, U.S. Government Printing Office, Washinton, D.C., 167 pp.

Jardín Botánico del Quindío (2013). Colección Nacional de Palmas de Colombia. 328 Registros, aportados por Manrique HF (Publicador, Proveedor de los Metadatos, Proveedor de Contenido), Bernal R (Investigador Principal), Instituto Alexander von Humboldt (Custodio de los datos). En línea, http://ipt.sibcolombia.net/rnjb/resource.do?r=coleccion-palmas-colombia, publicado el 19/04/2013., http://ipt.sibcolombia.net/rnjb/resource.do?r=coleccion-palmas-colombia

Lund Botanical Museum (LD): Lund Botanical Museum (LD)

Ceballos, G. y J. Ramírez Ruiz. 1997. Programa de erradicación de los roedores introducidos en la Isla Rasa, Baja California: un plan de restructuración ecológica. Universidad Nacional Autónoma de México. Instituto de Ecología. Bases de datos SNIB2010-CONABIO proyecto No. C004. México, D.F.

Fundación Estación Biológica Guayacanal (2013). Parcela Permanente en el Pantano Martos, 45 Registros, aportados por Vargas K ( Publicador, Creador del Recurso, Proveedor de los Metadatos, Autor), Ramirez W (Contacto del Recurso), Calderon MJ (Autor), Pinto J (Autor), Camargo G (Autor), En línea, http://ipt.sibcolombia.net/sib/resource.do?r=guayacanal-parcelasmartos, publicado el 14/03/2013., http://ipt.sibcolombia.net/sib/resource.do?r=guayacanal-parcelasmartos

Dávila Aranda, P. 1998. Flora Novo Galiciana-Gramineae. Universidad Nacional Autónoma de México. Instituto de Biología. Bases de datos SNIB2010-CONABIO proyecto No. F005. México, D.F.

Herbarium Hamburgense: HBGSpermatophyta - Herbarium Hamburgense

Herbario de la Universidad de Arizona, EUA

Plant Breeding and Acclimatization Institute (IHAR) - National Research Institute: Polish gene bank – passport data of plants accessions which are important in human life

WAHerb

González Elizondo, M. S., González Elizondo, M., Herrera Arrieta, Y. López Enriquez y J. A. Tena Flores. 1998. Base de datos sobre la flora de Durango. Instituto Politécnico Nacional. Centro Interdisciplinario de Investigación para el Desarrollo Integral Regional-Durango. Bases de datos SNIB2010-CONABIO proyecto No. P005. México, D.F.

Royal Botanic Garden Edinburgh. (2013) Living Collections Database

Pinos del Noreste de México (UANL)

Museum für Naturkunde Berlin: Neptune Deep-Sea Microfossil Occurrence Database

Verleyen E of Ugent and Vyverman W of Ugent.

Global Lacustrine Diatom Data.

Department of Plant Biology. Faculty of Biological Sciences. Univ. Murcia: Universidad de Murcia, Dpto. Biología Vegetal (Botánica), Murcia: MUB-MUSCI

Solano Camacho, E. 2002. Sistemática del género Polianthes L (Agavaceae). Universidad Nacional Autónoma de México. Facultad de Estudios Superiores Zaragoza. Bases de datos SNIB2010-CONABIO proyecto No. H230. México, D.F.

Staatliche Naturwissenschaftliche Sammlungen Bayerns: The Diatom Collection of Franz Josef Weinzierl at the Botanische Staatssammlung München

Salas Morales S. H. y A. Nava Zafra. 2007. Composición florística del Parque Nacional Huatulco. Sociedad para el Estudio de los Recursos Bióticos de Oaxaca, A. C. (SERBO). Bases de datos SNIB2010-CONABIO proyecto No. BK004. México, D.F.

Oleoducto Bicentenario (2013). RESCATE DE EPÍFITAS OLEODUCTO BICENTENARIO, TRAMO ARAGUANEY - BANADÍA (FOROFITOS) 215. Registros, aportados por Alejandro Calderón (Publicador, Proveedor de los Metadatos, Proveedor de Contenido, Creador del Recurso). En linea, http://ipt.sibcolombia.net/sib/resource.do?r=epifitas_forofitos, publicado el 08/05/2013.

SysTax: SysTax - Zoological Collections

GBIF-Sweden: Gothenburg Herbarium - Types (GBIF:IH:GB:Herbarium)

León Tejera, H. 2006. Inventario ficoflorístico de las comunidades arrecifales del Parque Nacional Bahías de Huatulco, Oaxaca. Universidad Nacional Autónoma de México. Facultad de Ciencias. Bases de datos SNIB2010-CONABIO proyecto BE020. México, D.F.

Bonilla-Barbosa, J. R. y J. Viana 2002. Flora acuática vascular de las regiones hidrológicas R66 (Lagos Cráter del Nevado de Toluca) y R67 (Río Amacuzac-Lagunas de Zempoala), México. Universidad Autónoma del Estado de Morelos. Centro de Investigaciones Biológicas. Bases de datos SNIB2010-CONABIO proyectos No. S058 y H141. México, D.F.

Marie-Victorin Herbarium (MT) from University of Montreal Biodiversity Centre. http://dx.doi.org/10.5886/rzav8bu2 (accessed on [date])., doi:10.5886/rzav8bu2

CLIMAP Project Members, (1997): Nannofossil assemblage in surface sediments, doi:10.1594/PANGAEA.51924

Herbario Kew del Real Jardín Botánico (RBGKEW)

Senckenberg: Collection Aves (bird skeletons) SMF

León Cortés, J. L. 2005. Patrones de diversidad florística y faunística del área focal Ixcan, selva Lacandona, Chiapas. El Colegio de la Frontera Sur. Unidad San Cristóbal de las Casas. Bases de datos SNIB2010-CONABIO. Plantas vasculares. Proyecto No. Y036. México, D.F.

Museo Argentino de Ciencias Naturales: Herbario Nacional de Plantas Celulares - Museo Argentino de Ciencias Naturales 'Bernardino Rivadavia'

University of Turku: Schulman's Neotropical Adelobotrys database

Pizarro, JosÃ© et al. (2005), MAF Lichen collection online database, UCM

Morejón, R., Sánchez, C., Regalado, L., Hernández, A. et Daniel A. 2006. Pteridophyte collection online database.

For citation format please consult http://www.nbi.noaa.gov.

Varied sources. Individual references may be obtained from website (paleodb.org)

iNaturalist.org: iNaturalist research-grade observations

Valencia, A. S. y G. Flores-Franco. 2006. Catálogo de Autoridad Taxonómica del género Quercus, Fagaceae en México.Herbario FCME, Facultad de Ciencias, UNAM. Base de Datos SNIB2010-CONABIO proyecto CS008. México, D.F.

Arías Montes, S. 2006. Evaluación del estatus de los géneros Pereskiopsis y Pereskia (Cactaceae) en los Apéndices de la CITES. Universidad Nacional Autónoma de México. Instituto de Biología. Base de datos SNIB2010-CONABIO proyecto No. ES002. México, D.F.

Crux, Jason A (1991): (Table 4) Distribution of Cretaceous calcareous nannofossils in ODP Hole 114-700B, doi:10.1594/PANGAEA.755411

Hernández Sandoval, L. G. 1998. Diversidad florística y endemismo en la Reserva de la Biósfera El Cielo, Tamaulipas, México. Universidad Autónoma de Tamaulipas. Instituto de Ecología Aplicada. Bases de datos SNIB2010-CONABIO proyecto No. P023. México, D.F.

Botanical Research Institute of Texas: Andes to Amazon Biodiversity Program

Museum für Naturkunde Berlin: MfN - Fossil plants (Mesophytic)

Árboles de la Península de Yucatán, Flora del Distrito de Tehuantepec, Oaxaca y Familia Asteraceae en México (IBUNAM)

Cleef, A. 1981. The vegetation of the Páramos of the Colombian Cordillera Oriental. PhD Thesis. Utrecht University.

Sturm H., O. Rangel. 1985. Ecologia de los Paramos Andinos: una visión preliminar integrada. Instituto de Ciencias Naturales - Universidad Nacional de Colombia. Bogota. 292p., n/a

TELDAP: Plantae, TAIF (Taiwan e-Learning and Digital Archives Program, TELDAP)

Colecciones de George Boole Hinton depositadas en el herbario de Kew: Familia Leguminosae

JBRJ - Instituto de Pesquisas Jardim Botânico do Rio de Janeiro. Jabot - Banco de Dados da Flora Brasileira. Disponível em: [http://www.jbrj.gov.br/jabot]., http://www.jbrj.gov.br/jabot/formularios/comocitar_jabot.php

Regalado, L., Lóriga J., Morejón, R., Hechavarría, L., Fuentes, I., Hernández A., Daniel, A., Caluff, M., Ventosa, I., Vale, A. et Echevarría R. 2006. Pteridophyte collection online database.

Herrera Silveira, J. A. 1999. Patrones de variación espacial y temporal de la biodiversidad fitoplanctónica de los cenotes abiertos de Yucatán. Instituto Politécnico Nacional. Centro de Investigación y de Estudios Avanzados-Mérida. Bases de datos SNIB2010-CONABIO proyecto No. M011. México, D.F.

Field Museum: Field Museum of Natural History (Botany) Bryophyte Collection

Basov, Ivan A; Krasheninnikov, Valery A (1983): (Figure 6) Distribution of benthic foraminifers in middle-upper and Quaternary in DSDP Site 71-512, doi:10.1594/PANGAEA.232392

Ortega Escalona, F. 1997. Computarización de la xiloteca Dr. Faustino Miranda del Instituto de Ecología, A.C. Instituto de Ecología, A.C. Bases de datos SNIB2010-CONABIO proyecto No. B201. México, D.F.

Staatliche Naturwissenschaftliche Sammlungen Bayerns: The Lichen Collection at the Botanische Staatssammlung München

Herbario Universidad de Málaga (MGC). Herbarium collections online databases.

Instituto de Investigação Científica Tropical: IICT Herbário LISC

Senckenberg: Collection Crustacea - ZMB

Programa de repatriación de datos de ejemplares mexicanos

Riba y Nava Esparza, R. 1998. Pteridoflora de Morelos. Universidad Autónoma Metropolitana. Unidad Iztapalapa. Bases de datos SNIB2010-CONABIO proyecto No. P141. México, D.F.

Botanical Garden, University of Valencia: Jardi Botanic de Valencia: VAL

The New York Botanical Garden: Herbarium of The New York Botanical Garden

PhytoKeys: Solanum_baretiae

Gonzales Chavez, Erasmo Isaias; Yepez Pinillos, Victor Eduardo (2007): Phytoplankton abundance in surface water in January 2007, Sechura Bay, Peru, doi:10.1594/PANGAEA.628527

Ríos Jara, E. y R. Ramírez Delgadillo, 2008. Inventario de la biota terrestre (Florístico) y marina (Invertebrados, peces y macroalgas bentónicos) del Parque Nacional Isla Isabel. Universidad de Guadalajara. Centro Universitario de Ciencias Biológicas y Agropecuarias. Bases de datos SNIB2010-CONABIO. Plantas. Proyecto No. BK018. México. D. F.

Fundación Alma (2013). Caracterización florística de las ciénagas en Gamarra y Barrancabermeja de 2012. 221 registros, aportados por Gutierrez-Camargo JC (Contacto del Recurso, Creador del Recurso), Cordoba-Sánchez MP (Autor), Garzón NV (Proovedor del Metadato, Publicador), En línea, http://ipt.sibcolombia.net/sib/resource.do?r=almacienagas2012, publicado el 22/03/2013, http://ipt.sibcolombia.net/sib/resource.do?r=almacienagas2012

Colunga-GarcíaMarín, P. 1997. Diversidad y conservación del germoplasma de henequén Agave fourcroyedes Lem. y su posible ancestro silvestre Agave angustifolia Haw. Centro de Investigación Científica de Yucatán A.C. Bases de datos SNIB2010-CONABIO proyecto No. B001. México, D.F.

Biebow, Nicole (2003): Assemblage of dinoflagellate cysts analysed in sediment core SO78-158-1, doi:10.1594/PANGAEA.126414

Instituto de Ciencias Naturales: Instituto de ciencias naturales

Bonilla Barbosa, J. R. 2007. Flora acuática vascular y de zonas inundables del área de protección de flora y fauna Laguna de Términos, Campeche, México. Universidad Autónoma del Estado de Morelos. Centro de Investigaciones Biológicas. Bases de datos SNIB2010-CONABIO proyecto No. BK031. México, D.F.

The U.S. Environmental Protection Agency through its Environmental Monitoring and Assessment Program (EMAP), http://www.epa.gov/emap/.

Chiang Cabrera, F. 2004. Inventario florístico de la región Calakmul-parte baja de la región Lacandona (Cuenca alta del Usumacinta y Marqués de Comillas). Universidad Nacional Autónoma de México. Instituto de Biología. Bases de datos SNIB2010-CONABIO proyecto No. Y004. México, D.F.

García Arévalo, A. 2000. Florística de la reserva de la biósfera de Mapimí. Instituto de Ecología A.C. Centro Regional-Durango. Bases de datos SNIB2010-CONABIO proyecto No. L035. México, D.F.

Cleef, A. 1981. The vegetation of the Páramos of the Colombian Cordillera Oriental. PhD Thesis. Utrecht University.

Abisaí Josué García Mendoza, FLORAOAXACA, Portal UNIBIO, Instituto de Biología, Universidad Nacional Autónoma de México, http://www.unibio.unam.mx consultada el dd/mm/yy.

Burckhardt D. Swiss Psyllid (Hemiptera) Collections - Geneva. Muséum d'histoire naturelle de la Ville de Genève

Hernández, F. J. et al. (2013). Bases de datos on-line de la Colección de Briófitos del Herbario de la Universidad de Salamanca: Briófitos (SALA-BRYO). (Date when consulted).

Téllez Valdés, O. 1998. Inventario florístico y base de datos de la Reserva Ecológica Sierra de San Juan, Nayarit, México. Universidad Nacional Autónoma de México. Instituto de Biología. Bases de datos SNIB2010-CONABIO proyecto No. P083. México, D.F.

Conservation International: Rapid Assessment Program (RAP) Biodiversity Survey Database

Herrera, L., Escribano, R. (2006)

AAU Herbarium Database

Vibrans Lindemann, H. 2003. Plantas exóticas del centro de México y obtención de imágenes para una flora virtual de malezas. Colegio de Postgraduados. Bases de datos SNIB2010-CONABIO proyecto No. U019. México, D.F.

Biebow, Nicole (2003): Assemblage of dinoflagellate cysts analysed in sediment core SO78-173-3, doi:10.1594/PANGAEA.126416

MEXUCH, Portal UNIBIO, Instituto de Biología, Universidad Nacional Autónoma de México, http://www.unibio.unam.mx consultada el dd/mm/yy.

Navarro Sigüenza, A. G. y J. A. Meave de Castillo. 1998. Inventario de la biodiversidad de vertebrados terrestres de los Chimalapas, Oaxaca. Universidad Nacional Autónoma de México. Facultad de Ciencias. Bases de datos SNIB2010-CONABIO. No. B002. México, D.F.

Herrera Arrieta, Y. 2004. Estudio taxonómico y base de datos del género Muhlenbergia de México. Instituto Politécnico Nacional. Centro Interdisciplinario de Investigación para el Desarrollo Integral Regional-Durango. Bases de datos SNIB2010-CONABIO proyecto No. V024. México, D.F.

University of Gdańsk, Dept. of Plant Taxonomy and Nature Conservation: Orchid Herbarium Collection

Dávila Aranda, P. 1998. Flora del Valle de Tehuacán-Cuicatlán: II fase. Universidad Nacional Autónoma de México. Instituto de Biología. Bases de datos SNIB2010-CONABIO proyecto No. F028. México, D.F.

Herrera Arrieta, Y. 1997. Estudio biosistemático del género Bouteloua (Poaceae) en México. Instituto Politécnico Nacional. Centro Interdisciplinario de Investigación para el Desarrollo Integral Regional-Durango. Bases de datos SNIB2010-CONABIO proyecto No. B061. México, D.F.

Pando, F. et al. (2003). MA Cryptogamic collections online databases. http://www.rjb.csic.es/herbario/crypto/crydb.htm. (date when consulted)

Habib, Daniel; Hayes, Dennis E; Pimm, Anthony C (2005): Dinoflagellate abundance of Hole 14-144, doi:10.1594/PANGAEA.249927

Búrquez Montijo, J. A. 1999. Diversidad vegetal en un gradiente en la Sierra Madre Occidental: flora y vegetación de la Región de San Javier y Yécora, Sonora. Universidad Nacional Autónoma de México. Instituto de Ecología. Bases de datos SNIB2010-CONABIO. Proyecto No. H122. México, D.F.

Chaisson, William P; Keigwin, Lloyd D; Rio, Domenico; Acton, Gary D; Shipboard Scientific Party, (2005): Range table from planktonic foraminifers in Hole 172-1064A, doi:10.1594/PANGAEA.315518

Rendón Aguilar, B. y J. Núñez Farfán. 1999. Flora útil del Municipio de la Huerta, Jalisco. Universidad Nacional Autónoma de México. Instituto de Ecología. Bases de datos SNIB2010-CONABIO proyecto No. L255. México, D.F.

IRD - Institute of Research for Development: Herbier de la Guyane

Castillo Campos, G. 2000. Diversidad y riqueza vegetal de los substratos rocosos del centro del estado de Veracruz. Instituto de Ecología A.C. Bases de datos SNIB2010-CONABIO proyecto No. L228. México, D.F.

Natural History Museum, Vienna - Herbarium W: Natural History Museum, Vienna - Herbarium W

MICROBIS

Botanic Garden and Botanical Museum Berlin-Dahlem: Staatliches Museum für Naturkunde Stuttgart, Herbarium

Jahn, R. & Kusber, W.-H. 2002 - (continuously updated): AlgaTerra Types - Information System on terrestrial and limnic Micro Algae.

Leya, T. (Ed.) 2013 - (periodically updated): Catalogue of the live Culture Collection of Cryophilic Algae contained at the Fraunhofer IBMT in Postdam-Golm.

Botanical Garden & Museum, Natural History Museum of Denmark: Botanical Museum, Copenhagen. Database of type specimens

Organization for Tropical Studies: Herbarium - Las Cruces Biological Station

University of Alberta Museums: University of Alberta Museums, Vascular Plant Herbarium

Röpert, D. (Ed.) 2000 - (continuously updated): Digital specimen images at the Herbarium Berolinense (B).

Vázquez, G. 2002. Diversidad y distribución de las comunidades de fitoplancton y peces de ríos y lagunas del volcán San Martín de la reserva de la biósfera Los Tuxtlas. Instituto de Ecología A.C. Bases de datos SNIB2010-CONABIO. Fitoplancton. Proyecto No. S022. México, D.F.

Organization for Tropical Studies: Wilson Botanical Garden - Las Cruces Biological Station

ACEDO C. et LLAMASZ F. -2008 onwards- LEB Vascular Plants Collections Online Databases. En la Web: http://www.gbif.es

Florida Museum of Natural History: invertebratezoology

Agentes Bioactivos de Plantas Desérticas de Latinoamérica (ICBG)

SysTax: SysTax - Herbaria

Herrera Silveira, J. A. 2002. Biodiversidad fitoplanctónica de cenotes: Patrones espaciales y temporales. Instituto Politécnico Nacional. Centro de Investigación y de Estudios Avanzados-Mérida. Bases de datos SNIB2010-CONABIO proyecto No. S004. México, D.F.

Contact Dave Watts for details on citation details.

Barajas Morales, J. 2001. Base de datos para la xiloteca del Instituto de Biología de la UNAM. Universidad Nacional Autónoma de México. Instituto de Biología. Base de datos SNIB2010-CONABIO proyecto No. T004. México, D.F.

Illinois Natural History Survey: Illinois Natural History Survey

Louisiana State University Herbarium, 2012, Department of Biological Sciences, http://www.herbarium.lsu.edu/

Biebow, Nicole (2003): Assemblage of dinoflagellate cysts analysed in surface sediment samples off Peru, doi:10.1594/PANGAEA.126413

Herbier des conservatoires et jardins botaniques de Nancy: Herbarium specimens

Álvarez Buylla, E. y F. Medina Freaner. 1997. Distribución, estructura poblacional y variación genética de algunas especies de pinos en peligro de extinción en México. Universidad Nacional Autónoma de México. Instituto de Ecología. Bases de datos SNIB2010-CONABIO proyecto No.B156. México, D.F.

Hágsater, E. y M. A. Soto. 1998. Diversidad y conservación de orquídeas de la región de Chimalapa, Oaxaca, México. Instituto Chinoin A.C. Bases de datos SNIB2010-CONABIO proyecto No. G024. México, D.F.

Staatliche Naturwissenschaftliche Sammlungen Bayerns: The Vascular Plant Collection at the Botanische Staatssammlung München

National Herbarium of New South Wales (NSW). Online plant information. http://plantnet.rbgsyd.nsw.gov.au

Real Jardín Botánico (CSIC): Real Jardin Botanico (Madrid), Vascular Plant Herbarium (MA)

Gómez Sánchez, M. 1997. Flora vascular del cerro El Zamorano. Universidad Autónoma de Querétaro. Facultad de Ciencias Naturales. Bases de datos SNIB2010-CONABIO proyecto No. L002. México, D.F.

British Antarctic Survey: ANTARCTIC PLANT DATABASE

Herbario IEB del Instituto de Ecología, A.C., México (IE-BAJíO)

US National Plant Germplasm System: United States National Plant Germplasm System Collection

Sturm H., O. Rangel. 1985. Ecologia de los Paramos Andinos: una visión preliminar integrada. Instituto de Ciencias Naturales - Universidad Nacional de Colombia. Bogota. 292p.

Botanical Garden & Museum, Natural History Museum of Denmark: Botanical Museum, Copenhagen, the Phycology Herbarium

Staatliche Naturwissenschaftliche Sammlungen Bayerns: The Fungal Collection at the Botanische Staatssammlung München

Martínez, M. 1998. Inventario florístico de la Sierra de San Carlos, Tamps. Universidad Autónoma de Tamaulipas. Instituto de Ecología Aplicada. Bases de datos SNIB2010-CONABIO proyecto No. P024. México, D.F.

Herrera Silveira, J. A. 1997. Biodiversidad de productores primarios de lagunas costeras del norte de Yucatán, México. Instituto Politécnico Nacional. Centro de Investigación y de Estudios Avanzados-Mérida. Bases de datos SNIB2010-CONABIO proyecto No. B019. México, D.F.

Pérez Farrera, M. A., Martínez Camilo. R., Meléndez López, E., Farrera Sarmiento, O. y H. Gómez Domínguez. 2004. Inventario florístico de la Frailescana (zona focal), Chiapas, México. Universidad de Ciencias y Artes de Chiapas. Escuela de Biología. Bases de datos SNIB2010-CONABIO proyecto No. Y012. México, D.F.

Schmook, B., Subproyecto Acahuales, En: Pozo de la Tijera, M del C y S. Calmé. 2005. Uso y monitoreo de los recursos naturales en el Corredor Biológico Mesoamericano (áreas focales Xpujil-Zoh Laguna y Carrillo Puerto). El Colegio de la Frontera Sur. Unidad Chetumal. Bases de datos SNIB2010-CONABIO proyecto No.BJ002. México, D.F.

Hágsater, E., Garcia-Cruz, J., Jiménez, R. y L. M. Sánchez. 1999. Estudio taxonómico-florístico de la familia Orchidaceae en el Bajío: tribus Epidendrae y Maxillariae. Instituto Chinoin A.C. Bases de datos SNIB2010-CONABIO proyecto No. H098. México, D.F.

Sir Alister Hardy Foundation for Ocean Science (SAHFOS): Continuous Plankton Recorder Dataset (SAHFOS)

ICoMM MICROBIS

Tiroler Landesmuseum Ferdinandeum: Tiroler Landesmuseum Ferdinandeum

Quero Rico, H. 2000. El complejo Brahea-Erythea (Palmae: Coryphoideae). Universidad Nacional Autónoma de México. Instituto de Biología. Bases de datos SNIB2010-CONABIO proyecto No. L216. México, D.F.

Bye Boettler, R. y M. Mendoza Cruz. 1998. Biodiversidad de Datura (Solanaceae) en México. Universidad Nacional Autónoma de México. Instituto de Biología. Bases de datos SNIB2010-CONABIO proyecto No.P088. México, D.F.

Cactáceas Columnares de México (IE-MORELIA, UNAM)

Flores Guido, J. S. 1999. Actualización del banco de datos florístico de la Península de Yucatán (BAFLOPY). Universidad Autónoma de Yucatán. Facultad de Medicina Veterinaria y Zootecnia. Bases de datos SNIB2010-CONABIO proyecto No. H146 y P112. México, D.F.

Fernández Nava, R., Reyes Toledo, B. y M. Casales Gómez. 2007. Computarización del Herbario ENCB, IPN. Fase IV. Base de datos de la familia Pinaceae y de distintas familias de la clase Magnoliopsida depositadas en el Herbario de la Escuela Nacional de Ciencias Biológicas, IPN. Instituto Politécnico Nacional. Escuela Nacional de Ciencias Biológicas. Bases de datos SNIB2010-CONABIO proyectos No. BC007. México, D.F.

Cárdenas Ramos, F. A. 1997. Catálogo para la utilización, conservación y disponibilidad de Phaseolus en México. Secretaría de Agricultura, Ganadería y Desarrollo Rural. Instituto Nacional de Investigaciones Forestales Agrícolas y Pecuarias. Bases de datos SNIB2010-CONABIO proyecto No. P047. México, D.F.

Herrera Arrieta, Y. 2009. Estudio florístico de las gramíneas de Zacatecas. Instituto Politécnico Nacional. Centro Interdisciplinario de Investigación para el Desarrollo Integral Regional-Durango. Bases de datos SNIB2010-CONABIO proyecto No. EE014. México, D.F.

Herrera Silveira, J. A. 1997. Biodiversidad de productores primarios de lagunas costeras del norte de Yucatán, México. Instituto Politécnico Nacional. Centro de Investigación y de Estudios Avanzados-Mérida. Bases de datos SNIB2010-CONABIO proyecto No. B019. México, D.F.

Herbarium (ALA), University of Alaska Museum, University of Alaska Fairbanks

García Mendoza, A. J. 1999. Revisión taxonómica del género Furcraea (Agavaceae) en México y Guatemala. Universidad Nacional Autónoma de México. Instituto de Biología Bases de datos SNIB2010-CONABIO proyecto No. H111. México, D.F.

Piontkovski, Sergey; Georgieva, LV; Senichkina, Ludmila G (2011): Phytoplankton abundance during Professor Vodyanitskiy cruise PV13, doi:10.1594/PANGAEA.757302

Ortiz-Pulido, R. 2006. Estudio de la avifauna y de las interacciones ave-planta en la Reserva de la Biosfera de la Barranca de Metztitlán Hidalgo, México. Universidad Autónoma del Estado de Hidalgo. Bases de datos SNIB2010-CONABIO proyecto No. AS010. México D. F.

Chiang Cabrera, F. 2007. Flora y datos básicos para la evaluación de las actividades apícola y forestal en tres áreas focales del corredor Sian Ka'an-Calakmul. Universidad Nacional Autónoma de México. Instituto de Biología. Bases de datos SNIB2010-CONABIO proyecto No. BE021. México, D.F.

Staatliche Naturwissenschaftliche Sammlungen Bayerns: The Exsiccatal Series "Triebel, Microfungi exsiccati"

GBIF-Sweden: Phanerogamic Botanical Collections (S)

Royal Botanic Gardens, Kew: Royal Botanic Gardens, Kew

Martin-Luther-University Halle-Wittenberg, The Erysiphales Collection at the University Halle-Wittenberg

Lira Saade, R. e I. Blanckaert. 2006. Estudio etnobotánico de las malezas útiles presentes en diferentes agroecosistemas en el municipio de Santa María Tecomavaca, Valle de Tehuacán-Cuicatlán, México. Universidad Nacional Autónoma de México. Facultad de Estudios Superiores Iztacala. Bases de datos SNIB2010-CONABIO. Proyecto No. BE010. México, D.F.

Nordic Genetic Resource Center (NORDGEN): Nordic Genetic Resources

Meave, J. A. 1998. Estudio de la diversidad florística en la región de la Chinantla, Sierra Norte de Oaxaca. Universidad Nacional Autónoma de México. Facultad de Ciencias. Bases de datos SNIB2010-CONABIO proyecto No. P069. México, D.F.

GBIF-Spain: Universidad Autónoma de Madrid, Biología, Acalypha

Kuhnt, Wolfgang; Kaminski, Michael A; Moullade, Michel (1989): Distribution of Upper Cretaceous deep water agglutinated foraminifera in sediments of the North Atlantic and its marginal seas (Tab. 1), doi:10.1594/PANGAEA.667860

Boisset, F. et al. (2009). VAL Cryptogamic collections online databases

Department of Organisms and Systems Biology. University of Oviedo: Universidad de Oviedo. Departamento de Biología de Organismos y Sistemas: FCO

Toledo Manzur, V. M. 2005. Potencial económico de la flora útil de los cafetales de la Sierra Norte de Puebla. Universidad Nacional Autónoma de México. Centro de Investigaciones en Ecosistemas. Bases de datos SNIB2010-CONABIO proyecto No. AE019. México, D.F.

Fernández Nava, R. 1997. Estudio monográfico de la familia Rhamnaceae en México. Instituto Politécnico Nacional. Escuela Nacional de Ciencias Biológicas. Bases de datos SNIB2010-CONABIO proyecto No. B059. México, D.F.

Administración de Parques Nacionales, Argentina: Registros biológicos en áreas protegidas obtenidos de documentos impresos

Paranaguá Bay – Plankton and Benthos Database

Philipps-Universität Marburg, The Fungal and Lichen Collections at the Herbarium Marburgense

Flores Guido, J. S. 1999. Actualización del banco de datos florístico de la Península de Yucatán (BAFLOPY). Universidad Autónoma de Yucatán. Facultad de Medicina Veterinaria y Zootecnia. Bases de datos SNIB2010-CONABIO proyecto No. H146 y P112. México, D.F.

Basov, Ivan A; Krasheninnikov, Valery A (1983): (Figure 7) Distribution of benthic foraminifers in the Oligocene to upper Miocene DSDP of Hole 71-513A, doi:10.1594/PANGAEA.232394

Corporación Autónoma para la Defensa de la Meseta de Bucaramanga, CDMB. Colección Biológica de Herbario Jardín Botánico Eloy Valenzuela. Floridablanca, 2013. 6821 Registros, aportados por Blanco DGA (Publicadora). En linea, http://ipt.sibcolombia.net/sib//resource.do?r=eloy_valenzuela, publicado el 20-05-2013., http://ipt.sibcolombia.net/sib//resource.do?r=eloy_valenzuela

Martín-Consuegra, E. et al. (2005). COA collections online databases.

Trujillo Jiménez, P., Quiroz-Castelán, H., Molina-Astudillo, F. I. y J. García-Rodriguez. 2002. Biodiversidad acuática del río Amacuzac, Morelos, México. Universidad Autónoma del Estado de Morelos. Centro de Investigaciones Biológicas. Bases de datos SNIB2010-CONABIO. Fitoplancton. Proyecto No. S150. México, D.F.

http://www.cpbr.gov.au/cpbr/herbarium

James Harris, et al., The Utah Valley State College Herbarium on-line catalog, http://herbarium.uvsc.edu, accessed on [day] [month] [year].

Chávez Rendón, C. 2006. Actualización e incremento del banco de datos de la colección de herbario del Jardín Etnobotánico de Oaxaca. Centro Cultural Santo Domingo. Bases de datos SNIB2010-CONABIO proyecto No. BC003. México, D.F.

Museo Argentino de Ciencias Naturales: Herbario Nacional de Plantas Vasculares - Museo Argentino de Ciencias Naturales 'Bernardino Rivadavia'

García Ruíz, I. 1999. Flora del Parque Nacional Pico de Tancítaro, Michoacán. Instituto Politécnico Nacional. Centro Interdisciplinario de Investigación para el Desarrollo Integral Regional-Michoacán. Bases de datos SNIB2010-CONABIO proyecto No. H304. México, D.F.

Jiménez Ramírez, J. 2001. Base de datos de las regiones prioritarias 113 y 120 en los municipios de Zirándaro y Coahuayutla, (Guerrero). Universidad Nacional Autónoma de México. Facultad de Ciencias. Bases de datos SNIB2010-CONABIO. Proyecto No. R177. México, D.F.

Skovmand, B. 1997. Colección, preservación y caracterización de cultivares criollos de origen español de trigo y centeno de México. Centro Internacional de Mejoramiento de Maíz y Trigo. Bases de datos SNIB2010-CONABIO proyecto No. E001. México, D.F.

Luna Vega, M. I. 2000. Florística y biogeografía de algunos bosques mesófilos de la Huasteca Hidalguense: Fase 3 (Chapulhuacán y Pisaflores). Universidad Nacional Autónoma de México. Facultad de Ciencias. Bases de datos SNIB2010-CONABIO. Proyecto No. L091. México, D.F.

Zamora Crescencio, P., Sánchez-González, Ma. C. y L. Aragón-Axomulco. 2005. Formación del banco de datos del herbario (UCAM). Universidad Autónoma de Campeche. Centro de Investigaciones Históricas y Sociales. Bases de datos SNIB2010-CONABIO proyecto No. AC002. México, D.F.

Bonilla Barbosa, J. R. 2004. Flora acuática vascular del área focal Felipe Carrillo Puerto, Corredor Biológico Sian Ka'an-Calakmul, Quintana Roo, México. Universidad Autónoma del Estado de Morelos. Centro de Investigaciones Biológicas. Bases de datos SNIB2010-CONABIO proyecto No. Y025. México, D.F.

Bioversity International: The System-wide Information Network for Genetic Resources (SINGER)

Utah State University: USU-UTC Specimen Database

Universidad de Antioquia (2012 - ). Registros biológicos del género Piper en el herbario HUA. 5600 Registros, aportados por Cardona-Naranjo F (Publicador, Autor), Calderón-Arias AM (Autor), Martínez-Figueroa YM (Autor), En línea, http://ipt.sibcolombia.net/sib/resource.do?r=genero_piper_hua,Versión 2 (última modificación en 22/11/2012)., http://ipt.sibcolombia.net/sib/resource.do?r=genero_piper_hua

Federación Nacional de Cafeteros de Colombia (2012). Primera caracterización biológica de plantas del proyecto "Incorporación de la Biodiversidad en el sector cafetero en Colombia. 3664 Registros, aportados por Gómez JA (Publicador, Proveedor de los Metadatos, Proveedor de Contenido), Vargas WG(Investigador Principal), Buitrago-Giraldo MC (Creador del Recurso, Procesador), León JA (Autor), Maya CA (Autor), Baquero-Rojas JC (Autor), Acosta DC (Autor), Arango-Díaz CA (Autor), Ortega-Giraldo JF (Autor), Benavides-Guzmán M (Autor), Escobar-Escobar DF (Autor), Santacruz M (Autor), En línea, http://ipt.sibcolombia.net/sib/resource.do?r=fnc_plantas_biodiversidad_sector_cafetero, publicado el 28/11/2012., http://ipt.sibcolombia.net/sib/resource.do?r=fnc_plantas_biodiversidad_sector_cafetero

University of Arizona Herbarium (ARIZ)

Biebow, Nicole (2003): Assemblage of dinoflagellate cysts analysed in sediment core SO78-175-1, doi:10.1594/PANGAEA.126417

GBIF New Zealand: New Zealand National Plant Herbarium (CHR)

Department of Organisms and Systems Biology. University of Oviedo: Universidad de Oviedo. Departamento de Biología de Organismos y Sistemas: FCO-Briof

Leibniz Institute of Plant Genetics and Crop Plant Research (IPK): IPK Genebank

Trujillo Jiménez, P. y J. A. Viana-Lases. 2002. Biodiversidad acuática del río Amacuzac, Morelos, México. Universidad Autónoma del Estado de Morelos. Centro de Investigaciones Biológicas. Bases de datos SNIB2010-CONABIO. Plantas vasculares. Proyecto No. S150. México, D.F.

Jahn, R. & Kusber, W.-H. 2005 - (continuously updated): AlgaTerra - Information System on Algae.

Field Museum: Field Museum of Natural History (Botany) Pteridophyte Collection

Senckenberg Museum für Naturkunde Görlitz, The Fungal Collection at the Senckenberg Museum für Naturkunde Görlitz

David Gernandt, PINACEAE, Portal UNIBIO, Instituto de Biología, Universidad Nacional Autónoma de México, http://www.unibio.unam.mx consultada el dd/mm/yy.

Department of Biology, University of Copenhagen: Galapagos grasses and sedges

Field Museum: Field Museum of Natural History (Botany) Fungi Collection

Sánchez Escalante, J. y L. Armenta. 2004. Actualización de la base de datos del Herbario de la Universidad de Sonora (USON). Universidad de Sonora. Bases de datos SNIB2010-CONABIO proyectos No. V051 y B047. México D. F.

Colección Plantas Vasculares, Herbario del Departamento de Biología, Bioquímica y Farmacia de la Universidad Nacional del Sur.

Botanical Research Institute of Texas: Botanical Research Institute of Texas

Martínez, M. 1999. Flora y vegetación de la Sierra de San Carlos en el municipio de San Nicolás, Tamaulipas. Universidad Autónoma de Querétaro. Facultad de Ciencias Naturales. Bases de datos SNIB2010-CONABIO proyecto No. L029. México, D.F.

Oleoducto Bicentenario (2013). RESCATE DE EPÍFITAS OLEODUCTO BICENTENARIO, TRAMO ARAGUANEY - BANADÍA (RESCATE) 915. Registros, aportados por Alejandro Calderón (Publicador, Proveedor de los Metadatos, Proveedor de Contenido, Creador del Recurso). En linea, http://ipt.sibcolombia.net/sib/resource.do?r=epifitas_oleoducto, publicado el 08/05/2013.

MNHN - Museum national d'Histoire naturelle: Cryptogams herbarium specimens

Colección de Flora Ficológica Marina de Tamaulipas, México (UANL)

Chávez León, G. 2006. Inventario florístico y faunístico del Parque Nacional Barranca del Cupatitzio, Michoacán. Secretaría de Agricultura, Ganadería, Desarrollo Rural, Pesca y Alimentación. Instituto Nacional de Investigaciones Forestales Agrícolas y Pecuarias. Bases de datos SNIB2010-CONABIO proyecto No. AS014. México, D.F.

Rangel-Ch., O and Ariza N. C. 2000. La vegetacion paramuna de los volcanes de Narino in Colombia Diversidad Biotica III, la región de Vida paramuna de Colombia. Rangel-Ch. J.O. 2000. Universidad Nacional de Colombia. Editorial Unibiblios. Bogotá, Colombia., ISBN 958-701-010-8

Federación Nacional de Cafeteros de Colombia (2013). Segunda caracterización biológica de plantas del proyecto "Incorporación de la Biodiversidad en el sector cafetero en Colombia. 5771 Registros, aportados por Gómez JA (Contacto del Recurso, Proveedor de los Metadatos, Proveedor de Contenido, Investigador Principal), Méndez-Vargas E(Autor), Buitrago-Giraldo MC (Creador del Recurso, Procesador, Publicador), En línea, http://ipt.sibcolombia.net/sib/resource.do?r=fnc_plantas_biodiversidad_sector_cafetero_2, publicado el 07/03/2013., http://ipt.sibcolombia.net/sib/resource.do?r=fnc_plantas_biodiversidad_sector_cafetero_2

ASCLEPIADACEAE, Portal UNIBIO, Instituto de Biología, Universidad Nacional Autónoma de México, http://www.unibio.unam.mx consultada el dd/mm/yy.

Lebgue Keleng, T. 2001. Flora de las Barrancas del Cobre. Universidad Autónoma de Chihuahua. Facultad de Zootecnia. Bases de datos SNIB2010-CONABIO. Proyecto No. R102. México, D. F.

Academy of Natural Sciences: MAL

Cleef A. 1977, 1983, 1984, 1989. Field Data.

Cleef. A.M. 1981. The vegetation of the paramos of the Colombian Cordillera Oriental. PhD Thesis. State University of Utrecht. 320pp. Also published as Dissertationes Botanicae, Baud 61, J. Cramer, Vadyz and "The Cuaternary of Colombia", Vol.9., n/a

Staatliches Museum für Naturkunde Karlsruhe, Fungus Collections at Staatliches Museum für Naturkunde Karlsruhe (Herbarium KR)

Netherlands Centre for Biodiversity Naturalis, section National Herbarium of the Netherlands: Nationaal Herbarium Nederland
